# Diversity of endophytic bacteria in mulberry (*Morus* spp.) scions with different genetic resources

**DOI:** 10.3389/fmicb.2025.1618773

**Published:** 2025-06-24

**Authors:** Yun-fei Zhang, Ying-ting Qin, Ze-yu Liu, Hao-ran Zheng, Xu-dong Hu, Xi-ling Wang

**Affiliations:** State Key Laboratory of Resource Insects, Colledge of Sericulture, Textile and Biomass Sciences, Southwest University, Chongqing, China

**Keywords:** diversity, endophytic bacteria, mulberry, graft, genetic resources

## Abstract

Endophytic bacteria in plants play crucial roles in promoting plant growth, facilitating nutrient acquisition, and enhancing stress tolerance. Although many studies have recently investigated endophytic bacteria in plants, the characteristics of endophytic bacterial communities in germplasm resource populations have rarely been reported. In this study, we investigated the endophytic bacterial communities of 21 mulberry scions, representing both wild and cultivated resources, all grafted onto a common rootstock and grown under identical cultivation conditions. High-throughput sequencing of 16S rRNA amplicons was performed using the Illumina MiSeq platform. The results revealed a total of 10 phyla, 31 classes, 50 orders, 50 families, and 113 genera of endophytic bacteria in the mulberry scions. The dominant phylum was Proteobacteria (89.07%), followed by Firmicutes (5.20%) and Actinobacteria (3.10%). At the genus level, *Sphingomonas* (32.84%), *Methylobacterium-Methylorubrum* (18.64%), and *Aureimonas* (8.76%) were the predominant genera enriched in the scion. Wild scions exhibited more complex endophytic bacterial communities compared to cultivated scions. Among the wild germplasm, XZBS and XZMK, originating from Tibet, China, displayed distinctive Actinobacteria signatures, suggesting a potential legacy of primitive geographic adaptation. Co-occurrence network analysis indicated that *Sphingomonas* and *Methylobacterium-Methylorubrum* acted as keystone taxa, forming critical bridges within the endophytic bacterial community network in the scions. Functional predictions further indicated that endophytic bacteria from wild species showed a greater metabolic capacity for aromatic compounds, amino acids, and carbohydrates compared with those from cultivated species. Moreover, analyses of the mulberry genetic population structure and endophytic bacterial community composition suggested that differentiation between wild and cultivated resources was associated with differences in endophytic bacterial communities. This study provides new insights into the diversity of endophytic bacteria among different mulberry germplasm resources and highlights geographically unique taxa, advancing our understanding of microbiome-driven adaptation in perennial grafted plants. It also offers a valuable reference for the future utilization of functional endophytic bacteria in mulberry improvement.

## Introduction

Endophytic plant bacteria (EPB) are microorganisms that reside within plant tissues during part or all of their life cycle without causing harm to the host. They can be isolated from plants through surface sterilization of tissues (Hallmann et al., 1997; dos Santos et al., 2022). As a group of plant growth-promoting bacteria (PGPB), EPB have shown significant potential in supporting green and sustainable agricultural practices. These bacteria contribute to plant nutrition by supplying essential elements such as nitrogen, phosphorus, and potassium through mechanisms like nitrogen fixation, phosphate solubilization, and potassium mineralization. Additionally, they promote plant growth by producing phytohormones such as auxins, cytokinins, and gibberellins, which regulate root development and shoot elongation. They also enhance plant stress tolerance by synthesizing antimicrobial compounds and activating induced systemic resistance (ISR), thereby priming plant defense responses. Furthermore, these microbes help shape the microbial community in ways that further support plant health and resilience (Poria et al., 2022; Stegelmeier et al., 2022). Such beneficial traits enable EPB to function as both biofertilizers (Seema et al., 2013; Nosheen et al., 2021) and biocontrol agents (Bacon et al., 2001; Khan et al., 2020), reducing chemical inputs, mitigating environmental impacts, and improving crop resilience. Advances in genome annotation and comparative genomics have further clarified the genetic basis of EPB’s plant-promoting properties. Whole-genome sequencing revealed that an endophytic bacterial strain, V4 isolated from tea, harbors genes responsible for indole-3-acetic acid (IAA) and siderophore synthesis, enhancing survival and host interactions (Jia et al., 2023). Similarly, an *Enterobacter* strain isolated from poplar was found to possess genes associated with plant niche adaptation and 4-hydroxybenzoate synthesis (Taghavi et al., 2010). These functional genes act as molecular bridges between EPB and their hosts, while the endophytic micro-ecosystem provides a foundation for functional implementation. Investigating these interactions remains a central focus in microbial ecology, especially given that most plants harbor endophytic bacteria across diverse tissue types (Afzal et al., 2019). Variations in endophytic bacterial communities influence the abundance of functional genes related to growth promotion and stress resistance. A well-balanced endophytic microecosystem not only fosters plant development but also strengthens its resilience to environmental stresses (Ali et al., 2021).

Research has shown that the community composition of EPB is shaped by a variety of factors, including host species (Ding and Melcher, 2016), genotype (Marques et al., 2015; De Almeida Lopes et al., 2016), plant organ (Hameed et al., 2015), developmental stage (Marques et al., 2015; Ren et al., 2015), season (Michalko et al., 2022), location (Ding and Melcher, 2016), soil type (Philippot et al., 2013), cultivation practices (Woźniak, 2019), host health (Bulgari et al., 2014), and fertilization (Kolb and Martin, 1988). For instance, urban tree studies highlighted seasonal and species-level differences in endophytic bacterial communities (Shen and Fulthorpe, 2015). In Stevia leaves, these communities varied across different growth stages (Yu et al., 2015). Nitrogen fertilization was also found to influence both the abundance and diversity of endophytic communities (Rodríguez-Blanco et al., 2015). The host genotype significantly impacted communities during early growth stages in sweet potato tuberous roots (Marques et al., 2015). Similarly, studies of soybean revealed significant differences in bacterial density between genotypes and tissues (De Almeida Lopes et al., 2016). In studies on grafted plants, it was found that the scion cultivar played a decisive role in shaping the composition of the leaf endophytic bacterial community in almond trees (Saldierna Guzmán et al., 2022). On rose, grafting altered the structure and function of the microbial community, and the genotype of the scion had a significant impact on the microbiome of the rootstock (Ramirez-Villacis et al., 2023).

Mulberry (*Morus* spp.), a traditional and important economic plant for sericulture, is widely distributed all over the world. China is one of the primary centers of origin (Yang and Hao, 2024). More than 3,000 mulberry germplasm resources, including wild and cultivated types have been collected from various regions and preserved by grafting propagation (Yu and Lou, 2016). There are significant differences in the morphological characters, economic traits, secondary metabolites and stress resistance of each resource. Molecular marker studies have shown that wild mulberry exhibits significantly higher genetic diversity than cultivated varieties (Zhao, 2005), providing a valuable reservoir of potentially functional endophytic bacteria. Researchers (Wu et al., 2018) reported that mulberry stems harbored abundant endophytic bacterial communities, and that the bacterial diversity in resistant varieties was higher than in susceptible ones. Recent studies have identified endophytic bacteria capable of antagonizing mulberry pathogens and promoting plant growth (Xu, 2020; Wang, 2021). In this study, 21 representative mulberry germplasm resources from East, Central, and Southwest China were selected according to genetic population structure to investigate the diversity and functional potential of endophytic bacterial communities in mulberry by Illumina MiSeq high-throughput sequencing of 16S rRNA amplicons. The findings offer valuable insights for integrating endophytic bacteria into sustainable mulberry cultivation practices. They also establish a foundation for future studies aimed at harnessing functional endophytes to enhance grafted mulberry resilience and productivity.

## Materials and methods

### Sample collection and surface sterilization

In June 2023, 21 samples of one-year-old mulberry branches were collected from the *Mulberry Origin* germplasm nursery (106°25′27.678′′E, 29°49′30.650′′ N) at Southwest University, Beibei District, Chongqing. Based on previous work on simplified genome sequencing of mulberry (unpublished) and traditional classification experience, the 21 samples were divided into two groups: Group A, which represents wild resources, and Group B, which comprises domesticated resources (Table 1). All samples were grafted onto the “Guisangyou 62” rootstock, grown in the same field, and subjected to the same natural climatic conditions. The rootstocks were managed using identical cultivation practices during the seedling stage. Except for the two samples (XZBS and XZMK), which were collected from Tibet, China, and grafted in 2019, all other samples were uniformly grafted in 2006. After grafting, all samples received identical management in terms of fertilization, irrigation, and pruning. For each mulberry resource, three distinct individual trees were selected as biological replicates. From each individual, three healthy one-year-old branches, each approximately 50 cm in length, were randomly selected and pooled to form one composite sample, in order to enhance the representativeness of each biological replicate. Summer pruning in the germplasm nursery is generally performed between May and June. The branches that grow after summer pruning until the next pruning season are considered one-year-old branches, which exhibit a largely consistent growth and development period. The leaves were removed, leaving the winter buds intact. After collection, the branches were temporarily stored in sterile bags at 4°C and processed for cutting and sterilization within 24 h. The branches were first rinsed with tap water, then trimmed into 2–3 cm segments using sterilized pruning shears. Prior to trimming each sample, the pruning shears were re-sterilized to minimize the risk of cross-contamination between samples. Additionally, all containers used during the surface sterilization procedure were pre-sterilized, and each sample was individually processed in a dedicated container under aseptic conditions. The trimmed segments were soaked and rinsed 3–4 times with sterile water, immersed in 75% ethanol for 30–40 s, rinsed again 2–3 times with sterile water, and soaked in 3.5–4% sodium hypochlorite solution (containing available chlorine) for 6 min (MACKLIN, Cat. No. S817441, Shanghai, China). Finally, they were rinsed 3–4 times with sterile water. The sterile water from the final rinse was plated onto Potato Dextrose Agar (PDA) and Luria-Bertani (LB) media to confirm sterility. Rootstock root samples were collected from the healthy underground portion, 5–20 cm below the soil surface, and surface sterilization was performed following the same protocol. The entire sterilization process was carried out under sterile conditions in a laminar flow hood. Soil samples were collected from the loose soil within a 30 cm radius of the rootstock root zone. All samples included three biological replicates and were stored at −80°C.

**Table 1:** Collection sites and years of all mulberry resources.

Resource ID	Group	Source location	Grafting time
G1	GroupA	Guizhou	2006
TQ29	GroupA	Sichuan	2006
TQ53	GroupA	Sichuan	2006
PS	GroupA	Sichuan	2006
DCYS	GroupA	Sichuan	2006
C5	GroupA	Sichuan	2006
XZBS	GroupA	Tibet Autonomous Region	2019
XZMK	GroupA	Tibet Autonomous Region	2019
TW	GroupA	Yunnan	2006
CK3	GroupA	Chongqing	2006
HNHS	GroupA	Hunan	2006
FY	GroupB	Zhejiang	2006
JH	GroupB	Zhejiang	2006
HCS	GroupB	Zhejiang	2006
DMD	GroupB	Zhejiang	2006
HS29	GroupB	Jiangsu	2006
ZD11	GroupB	Jiangsu	2006
HS192	GroupB	Jiangsu	2006
BS5	GroupB	Yunnan	2006
S7	GroupB	Chongqing	2006
XL20	GroupB	Hunan	2006

### DNA extraction and PCR amplification

Total genomic DNA samples were extracted using the OMEGA Soil DNA Kit (M5635-02) (Omega Bio-Tek, Norcross, GA, United States) according to the manufacturer’s instructions and stored at −20°C prior to further analysis. The quantity and quality of extracted DNA were measured using a NanoDrop NC2000 spectrophotometer (Thermo Fisher Scientific, Waltham, MA, USA) and agarose gel electrophoresis, respectively. Bacterial 16S rRNA genes were amplified using the primer pair (5′-AACMGGATTAGATACCCKG-3′ and 5′-ACGTCATCCCCACCTTCC-3′) (Beckers et al., 2016). The PCR reaction volume was 25 μL and contained 5 μL of 5 × reaction buffer, 5 μL of 5 × GC buffer, 2 μL of dNTPs (2.5 mM), 1 μL of forward primer (10 μM), 1 μL of reverse primer (10 μM), 2 μL of DNA template, 8.75 μL of ddH₂O, and 0.25 μL of Q5^®^ High-Fidelity DNA Polymerase (NEB-M0491L). PCR cycling conditions comprised an initial denaturation at 98°C for 3 min, 25–30 cycles of denaturation at 98°C for 15 s, annealing at 55°C for 30 s, and extension at 72°C for 30 s, followed by a single extension at 72°C for 5 min and a final hold at 10°C.

### Amplicon sequencing and processing of sequencing data

The amplification products were verified using agarose gel electrophoresis, and sequencing was performed by Personalbio (Shanghai, China) on the Illumina MiSeq platform. The raw sequencing data were stored in FASTQ format. The 16S rRNA sequencing data have been deposited in the NCBI Sequence Read Archive under the BioProject accession number PRJNA1255390. Data processing was carried out using QIIME2 (version 2019.4) (Bolyen et al., 2019) with slight modifications to the official tutorials.^1^ Briefly, raw sequence data were demultiplexed using the *demux* plugin, followed by primer trimming using the *cutadapt* plugin (Martin, 2011). Sequences were then quality filtered, denoised, merged, and checked for chimeras using the *DADA2* plugin (Callahan et al., 2016). To remove low-quality bases, the first 10 bases of both forward and reverse reads were trimmed using --p-trim-left-f 10 and --p-trim-left-r 10. Reads were then truncated at positions 250 (forward) and 200 (reverse) using --p-trunc-len-f 250 and --p-trunc-len-r 200, based on the per-base quality score profiles. Sequences containing ambiguous bases (Ns) or exceeding the expected error threshold were discarded. DADA2 performed denoising by constructing an error model from the data, correcting sequencing errors, and inferring exact amplicon sequence variants (ASVs). Chimeric sequences were identified and removed using the consensus-based method. Only non-chimeric, high-quality ASVs were retained for downstream taxonomic and diversity analyses. ASVs were aligned with *MAFFT* (Katoh et al., 2002) and used to construct a phylogeny with *FastTree2* (Price et al., 2010). The Greengenes database has been widely used in previous studies of microbial diversity. To facilitate comparison with earlier literature and information mining, taxonomic annotation of the ASVs was performed using this database (Release13.8^2^) (DeSantis et al., 2006). A phylogenetic tree was constructed using the maximum likelihood method implemented in *FastTree* (version 2.1.11^3^). Metabolic pathway and functional predictions were performed using *PICRUSt2* (Douglas et al., 2020). First, 16S rRNA gene sequences from known microbial genomes were aligned to construct a phylogenetic tree and infer gene functional profiles of common ancestors. The 16S rRNA feature sequences were then aligned with reference sequences to build a new phylogenetic tree. The *Castor* (Louca et al., 2018) hidden-state prediction algorithm was used to infer the closest species for the feature sequences based on the gene family copy numbers associated with reference sequences in the phylogenetic tree. By integrating the abundance of feature sequences in each sample, the gene family copy numbers for each sample were calculated. Finally, the annotation results from the MetaCyc,^4^ KEGG,^5^ and COG^6^ databases were used to obtain the metabolic pathway abundance profiles of each sample.

### Bioinformatics and statistical analysis

The bacterial relative abundance, α-diversity, community composition, β-diversity, and functional analyses were performed using the Personalbio online cloud platform.^7^ The α-diversity indices, including Chao1, Shannon, Faith’s PD, Pielou’s evenness, and Good’s coverage, were visualized as boxplots. Group comparisons of alpha diversity indices were conducted using the Kruskal-Wallis test due to the non-normal distribution of diversity metrics. All *p*-values were adjusted for multiple comparisons using the Benjamini-Hochberg method to control the false discovery rate (FDR). β-diversity was illustrated using Non-Metric Multidimensional Scaling (NMDS) based on abundance distance matrices, with weighted UniFrac and unweighted UniFrac distance algorithms calculated separately. Weighted and unweighted UniFrac distances were used to capture both abundance-driven and presence/absence-based community differences, respectively. LEfSe and ALDEx2 analyses were employed to identify significantly different microbial taxa. The LEfSe analysis was conducted using the online platform provided by Personalbio, with an LDA score > 3.0 and a *p*-value < 0.05 as thresholds for statistical significance. Differential abundance analysis was performed using R package ALDEx2 (version 1.34.0) (Fernandes et al., 2013). Raw count data were first subjected to centered log-ratio (clr) transformation. Statistical testing was conducted based on 128 Monte Carlo resamplings. Welch’s *t*-test was applied for pairwise comparisons, and the Kruskal–Wallis test was used for multiple group comparisons. Effect size was calculated for two-group comparisons. Features with Benjamini–Hochberg FDR-adjusted *p*-values < 0.05 and effect sizes greater than 1 (for two-group comparisons) were considered significantly differentially abundant. LEfSe and ALDEx2 analyses were jointly applied to identify differentially abundant microbial features. LEfSe was used to highlight differential taxa at various taxonomic ranks, while ALDEx2 was employed to detect differences at the ASV level. Some ASVs were identified using sequence alignment with BLAST on the NCBI online platform^8^ and the *blastn* tool (version 2.16.0) (Camacho et al., 2009). Multiple sequence alignment and phylogenetic tree construction were performed using MEGA-cc (version 11.0.13), and tree visualization was optimized using the iTOL platform.^9^ Bar plots, heatmaps, Venn diagrams, and MA plots were generated using Prism and the CNS online platform,^10^ with *p*-values < 0.05 considered statistically significant. Microbial co-occurrence network analysis was constructed using Python (3.12.4), and the results were visualized with Cytoscape (version 3.10.3). The genetic evolutionary relationships of the host samples were determined based on simplified genome sequencing results. The sequencing data were aligned to the reference genome using the MEM algorithm of BWA (version 0.7.15-r1140) (Li and Durbin, 2009). Subsequently, GATK (version 3.7) (McKenna et al., 2010) was used for joint variant calling, including SNP and InDel detection. Finally, a phylogenetic tree was constructed using the neighbor-joining method implemented in MEGAX (Kumar et al., 2018). The grafting schematic diagram was created using the online tool Figdraw.^11^ Species abundance information (Supplementary Tables S1–S3) and EC metabolic pathway abundance information were provided in Supplementary Tables S4–S6.

## Results

### Sequence data and phylum-level analysis

**Figure 1:**
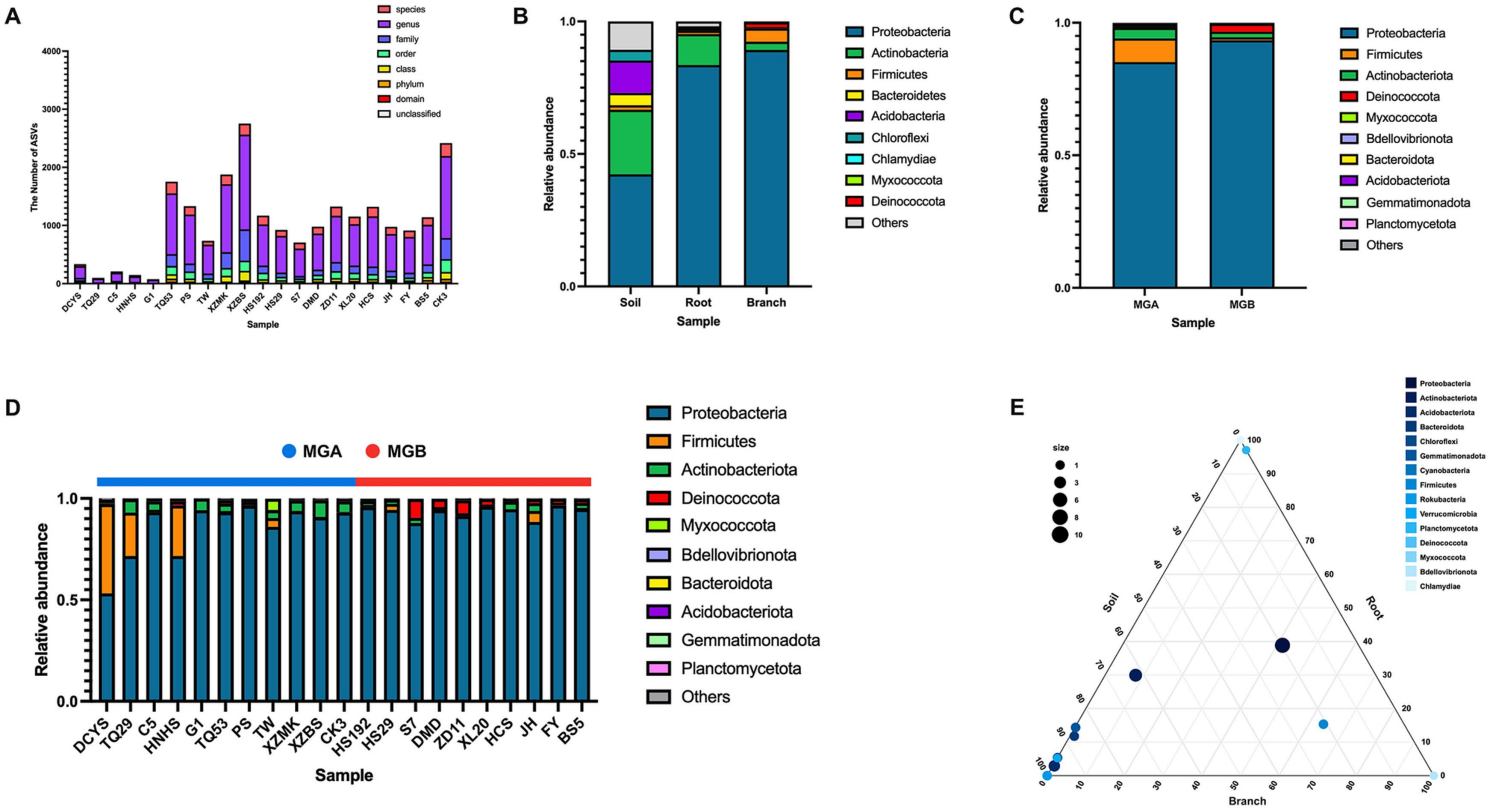
Microbial composition analysis at the phylum-level. **(A)** Taxonomic annotation of species. The horizontal axis represents individual branch samples, and the vertical axis represents the number of ASVs/OTUs annotated at the highest taxonomic level in each sample. **(B)** Relative abundance from different habitats. **(C)** Relative abundance from different host groups. **(D)** Relative abundance from different branch samples. **(E)** Ternary plot. Each side of the triangle represents a habitat. The position of each point indicates the compositional ratio of a phylum across the three habitats, and the size of each point reflects the relative abundance of that phylum.

After quality filtering, a total of 4,023,912 high-quality sequences were retained from 21 one-year-old branches with an average read length of 376.77 bp (Supplementary Table S7). Based on 100% similarity, 44,167 ASVs of endophytic bacteria were identified from the V5–V7 region of the effective sequences in the one-year-old branch samples. A total of 1,730 and 12,646 ASVs were obtained from the rootstock root and soil samples, respectively. Rarefaction curves for all samples reached a plateau (Supplementary Figure S1), indicating that the sequencing depth was sufficient to capture most bacterial community characteristics. Figure 1A showed that samples DCYS, C5, TQ29, HNHS, and G1 had fewer than 500 ASVs, with G1 exhibiting the lowest count (<100). TQ53, XZMK, XZBS, and CK3 had more than 1,500 ASVs, with XZBS having the highest count (>2,500). ASV counts for the other samples ranged from 1,000 to 1,500. ASV numbers showed greater variation among wild samples (MGA), whereas cultivar samples (MGB) had more consistent ASV counts. The population classification results revealed that the ASVs in the one-year-old branches belonged to 10 phyla, 31 classes, 50 orders, 50 families, and 113 genera. The dominant bacterial groups (with relative abundance greater than 1%) in the branches were Proteobacteria (89.07%), Firmicutes (5.20%), Actinobacteriota (3.10%), and Deinococcota (1.79%). In the rootstock roots, the dominant phyla were Proteobacteria (83.52%), Actinobacteriota (11.73%), and Firmicutes (1.25%). In the soil, the dominant phyla included Proteobacteria (42.31%), Actinobacteriota (24.34%), Acidobacteriota (12.25%), Bacteroidota (4.66%), Chloroflexi (4.06%), Gemmatimonadota (2.20%), Cyanobacteria (2.03%), Firmicutes (1.70%), Rokubacteria (1.57%), and Verrucomicrobia (1.09%) (Figure 1B; Supplementary Table S8). The relative abundance of Proteobacteria was lower in MGA than in MGB, whereas Firmicutes were more abundant in MGA. Deinococcota exhibited a higher abundance in MGB (Figure 1C; Supplementary Table S9). The relative abundance of Firmicutes in DCYS, TQ29, and HNHS, Deinococcota in S7, ZD11, Myxococcota in TW, and Actinobacteriota in XZBS were significantly higher compared to other resources (Figure 1D). The distribution patterns of dominant taxa across three different habitats were identified by a ternary plot (Figure 1E). Proteobacteria, Actinobacteriota, and Firmicutes were highly abundant in all three habitats. Bacteroidota, Chloroflexi, Gemmatimonadota, and Cyanobacteria were predominantly distributed in the soil, while Deinococcota, Myxococcota, and Bdellovibrionota were more prevalent in the branches. The relative abundance of Actinobacteriota, Acidobacteriota, and Bacteroidota gradually decreased, while Proteobacteria and Firmicutes gradually increased with the habitat transition from soil to rootstock roots and then to one-year-old branches. Although this variation is the primary trend across all samples, the community characteristics in the branches ultimately differ due to differences in genotype and resource origin, even under the same rootstock and soil environmental conditions.

### Genus-level analysis and shared species analysis

**Figure 2:**
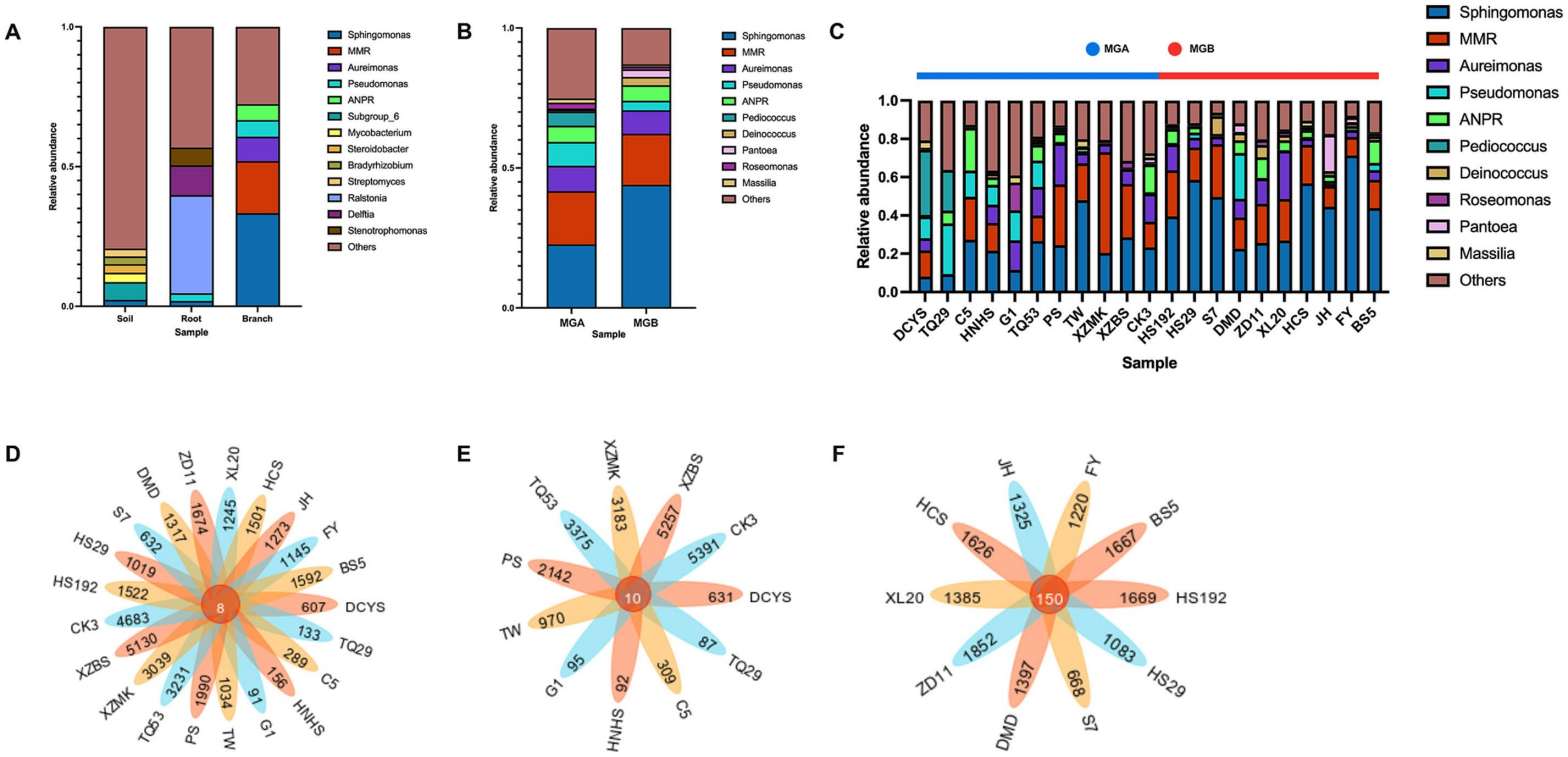
Genus-level analysis and shared species analysis. **(A–C)** Represent the genus-level composition of endophytic bacterial communities in different samples and groups. **(D)** Represents the number of ASVs shared among all 21 samples. **(E)** Represents the number of ASVs shared within Group A. **(F)** Represents the number of ASVs shared within Group B.

The relative abundance of genera in the mulberry branches, rootstocks, and soil was shown in Figure 2A (Supplementary Table S10). The *Sphingomonas* (32.84%), *Methylobacterium-Methylorubrum* (MMR, 18.64%), *Aureimonas* (8.76%), *Pseudomonas* (6.08%), and *Allorhizobium-Neorhizobium-Pararhizobium-Rhizobium* (ANPR, 5.68%) were dominant genera of endophytes in mulberry branches. *Ralstonia* (35.13%), *Delftia* (10.67%), and *Stenotrophomonas* (6.34%) were dominant genera in the rootstock roots. Most of the bacterial genera in the soil could not be identified. *Sphingomonas* and *Pseudomonas* were found to gradually increase in abundance from the soil to the roots and then to the branches, with *Sphingomonas* particularly accumulating in the branches to become the dominant taxon. In the branches, the composition of the top five genera in terms of relative abundance was similar between wild (MGA) and cultivated (MGB) hosts. *Sphingomonas* accounted for 22.70% in MGA, which was lower than in MGB (44.00%), whereas *Pseudomonas* was more abundant in MGA (8.52%) than in MGB (3.40%). The remaining genera, MMR, *Aureimonas*, and ANPR, showed no substantial differences between the two groups (Figure 2B; Supplementary Table S11). In addition, *Pediococcus* (5.03%), *Bacillus* (1.88%), *Ralstonia* (1.86%), and *Massilia* (1.54%) exhibited higher relative abundances in MGA compared to MGB. Conversely, *Deinococcus* (3.01%), *Pantoea* (2.71%), *Escherichia-Shigella* (1.11%), and *Paenibacillus* (0.84%) were more abundant in MGB than in MGA. Among the top ten genera by relative abundance, *Pseudomonas*, *Pediococcus*, *Deinococcus*, and *Pantoea* exhibited clear host preferences: *Pediococcus* was detected only in DCYS and TQ29, while *Pantoea* showed higher abundance in the JH samples (Figure 2C). Meanwhile, a large proportion of unannotated species were observed in MGA, indicating a knowledge gap in the identification of endophytic bacteria in wild mulberry resources. The number of common and unique bacterial ASVs in the different samples was presented in Venn diagrams. Only 8 shared ASVs were found across all mulberry germplasm resources (Figure 2D), potentially representing the core endophytic bacterial genera in the mulberry branches (Supplementary Table S12). Ten shared ASVs were detected in MGA (Figure 2E), while 150 were found in MGB (Figure 2F). The number of shared endophytic bacterial species within MGA, which exhibited greater genetic diversity, was much lower than that within MGB. This suggests that endophytic bacterial composition is influenced by the genotype of the scion.

### Diversity of endophytic bacterial communities with different mulberry genetic resources

**Figure 3:**
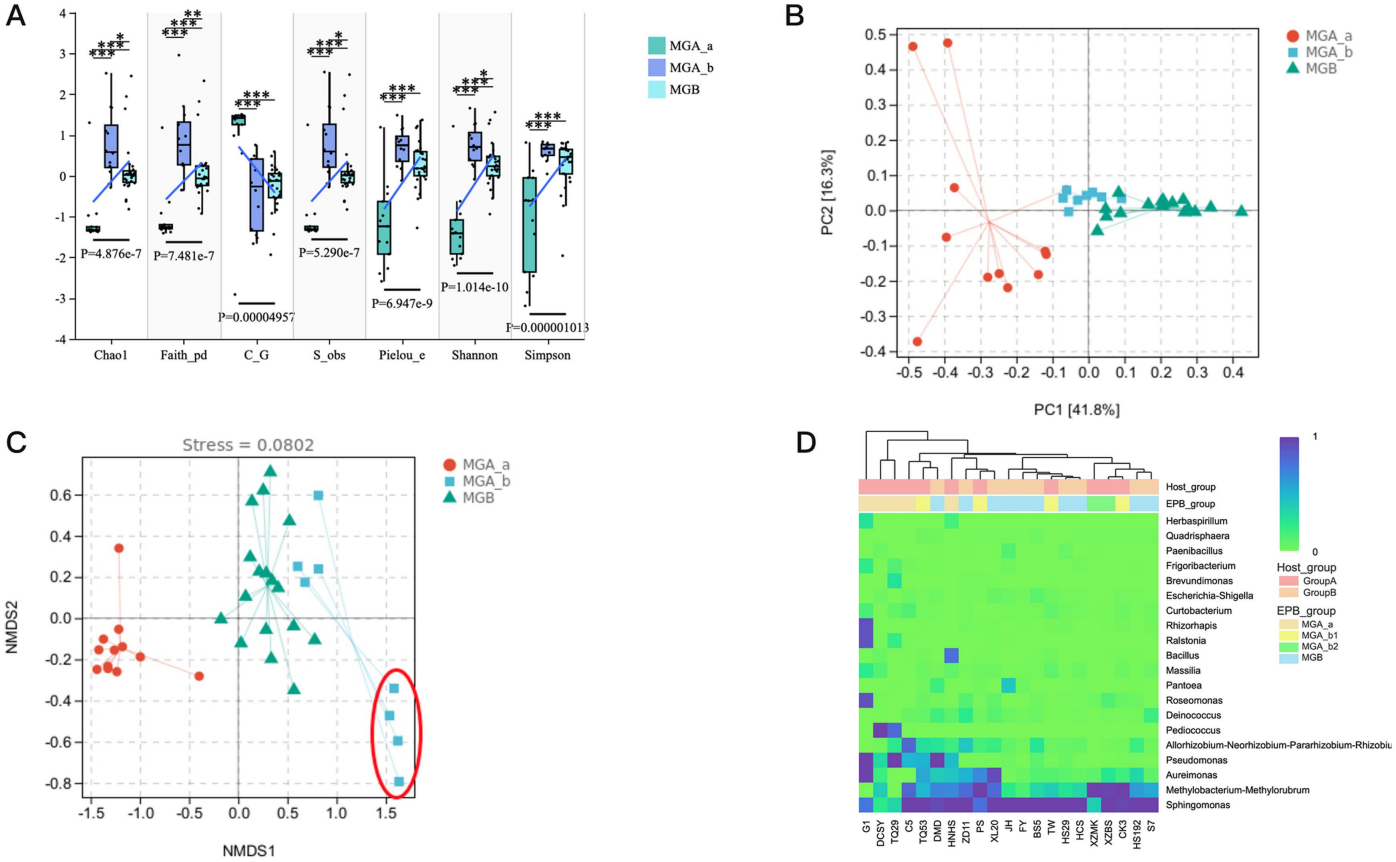
Diversity of endophytic bacterial communities with different mulberry genetic resources. **(A)** The x-axis represents different alpha diversity index, and the y-axis indicates the corresponding alpha diversity index values. The numbers below the diversity index labels denote the *p*-values from the Kruskal-Wallis test and using the Benjamini-Hochberg method to control the false discovery rate (FDR). **(B)** PCA plot, PC1 = 41.8%, PC2 = 16.3%. **(C)** Each point in the figure represents a sample, with different colors indicating different groups. The closer (or farther) the distance between two points, the smaller (or larger) the difference in microbial community composition between the corresponding samples. The unweighted UniFrac distance algorithm was used in the analysis. **(D)** Heatmap of species composition. The top 20 genera were clustered using UPGMA based on Euclidean distance of the species composition data and arranged according to the results of min-max normalization.

To comprehensively evaluate the alpha diversity of microbial communities, the Chao1 and Observed Species indices were used for richness, the Shannon and Simpson indices for diversity, Faith’s PD for phylogeny-based diversity, Palou’s index for evenness, and Good’s coverage for sequencing completeness. The results were shown in Supplementary Figure S2 (Supplementary Table S13). It was found that there were no significant differences among HS192, HS29, S7, DMD, ZD11, XL20, HCS, JH, FY, and BS5 (MGB). Similarly, no significant differences were observed among samples in MGA_a (DCYS, C5, TQ29, HNHS, and G1) or in MGA_b (TW, PS, TQ53, CK3, XZMK, and XZBS). However, there were significant differences among these sample groups. Therefore, the alpha diversity index was subdivided into three subgroups named MGA_a, MGA_b and MGB (Figure 3A; Supplementary Table S14) and significant differences were observed among the new subgroups. This result was consistent with the ASV number observed in the mulberry germplasm resources (Figure 1A). Principal component analysis (PCA) showed that PC1 (41.1%) was identified as the primary contributor to the observed differences with a clear separation among MGA_a, MGA_b and MGB (Figure 3B). A loading plot was used to assess the contribution values of key genera to community composition, and it was found that the main endophytic bacterial genera influencing the PC1 dimension were *Sphingomonas* and *Pseudomonas* (Supplementary Figure S3). NMDS analysis (unweighted UniFrac) showed that MGA_a was separated from MGA_b, whereas MGA_b clustered with MGB, except for XZMK and XZBS, which originated from Tibet (Figure 3C; Supplementary Figure S4). Therefore, we decided to group XZBS and XZMK into a new subgroup named MGA_b2, while the other samples originally in MGA_b were designated as MGA_b1 for subsequent analysis. A heatmap showed that MGA_a had a more diverse endophytic bacterial community compared to MGB, while the communities of MGA_b1 and MGA_b2 were more similar to MGB. With the transition from wild to cultivated resources, community diversity gradually decreased. *Sphingomonas* and MMR remained relatively abundant across all samples (Figure 3D).

### Specific microbial taxon of subgroups

**Figure 4:**
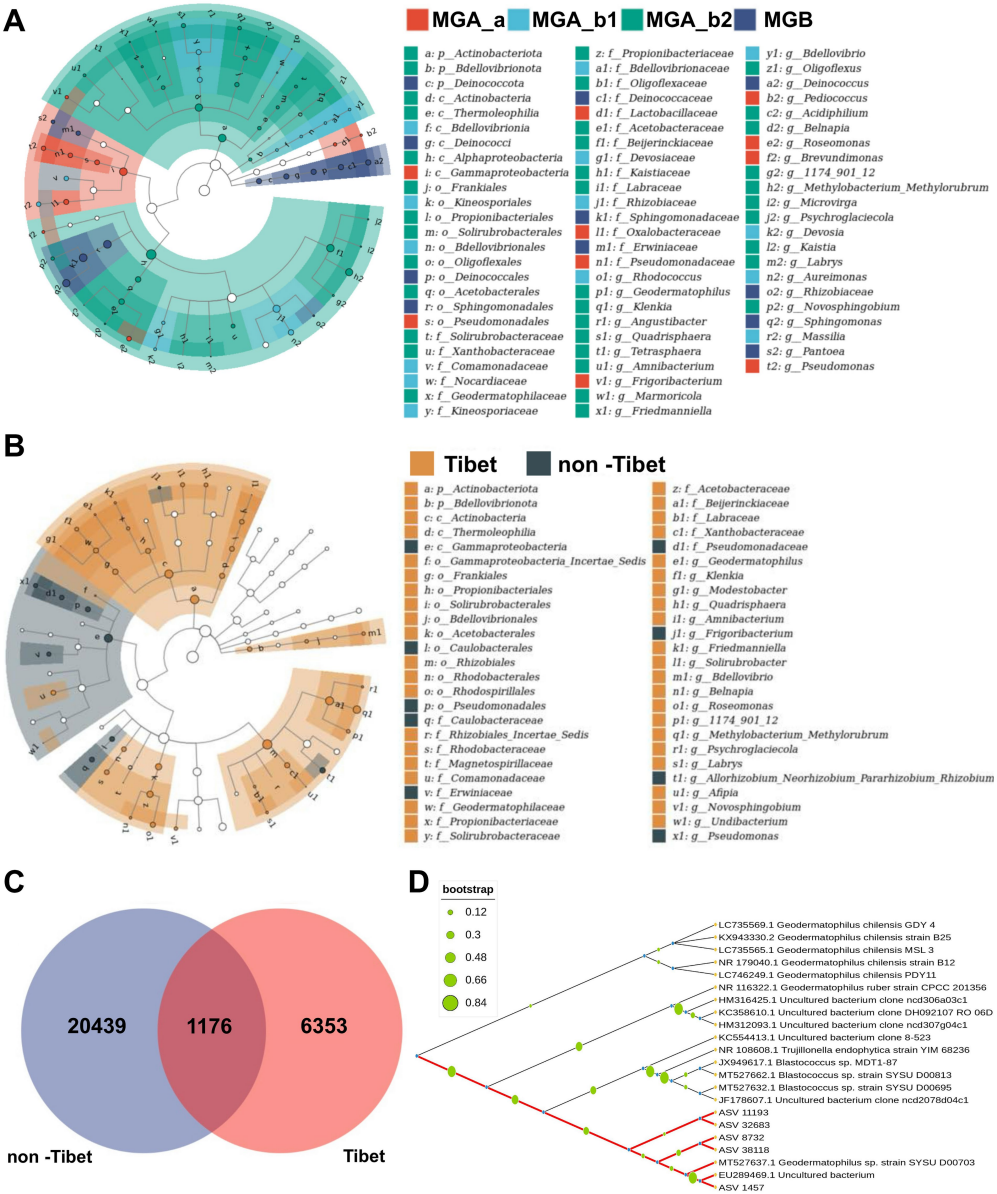
Specific microbial taxa of subgroup. **(A)** The LEfSe analysis of phylum. **(B)** The LEfSe analysis of genus. The size of each node corresponds to the mean relative abundance of the taxon. Hollow nodes represent taxa with no significant differences between groups, whereas colored nodes indicate taxa showing significant intergroup differences. The sectors in different colors represent different groups. The LDA score threshold was set at ≥ 3.0. **(C)** Venn diagram of regional groupings. **(D)** Phylogenetic tree of Actinobacteria unique in Tibet, constructed using maximum likelihood with 1,000 bootstrap replicates. Node sizes represent bootstrap support values.

LEfSe analysis was performed to identify the relevant endophytic bacteria taxa responsible for different subgroups (MGA_a, MGA_b1, MGA_b2, MGB). A total of 72 taxa were identified in this analysis including 3 phyla, 6 classes, 10 orders, 21 families, 32 genera. In MGA_a, the indicator species were primarily concentrated in Gammaproteobacteria (i), including Oxalobacteraceae (l1) and Pseudomonadaceae (n1). MGA_b1 was mainly represented by Rhizobiaceae (j1). MGA_b2 had the highest number of specific microbial taxa, primarily comprising multiple species from Actinobacteriota (a) and Alphaproteobacteria (h). MGB was represented by Deinococcota (c) and Sphingomonadales (r) (Figure 4A). The results showed that Actinobacteriota (a) was the most specific taxon of germplasm resources from Tibet, China. To further analyze the indicator species of endophytic bacterial communities from mulberry resources in the Tibet region, we categorized the samples into Tibet and non-Tibet groups. Figure 4B displayed that Actinobacteriota (a) and its classes Actinobacteria (c) and Thermoleophilia (d) were specific taxa in Tibet, China. Gammaproteobacteria (e) with Pseudomonadales (p) were the most specific taxa in non-Tibet regions. Figure 4C showed that a total of 1,176 endophytic bacterial ASVs were shared, with 6,353 ASVs unique to the Tibet region and 20,439 ASVs unique to non-Tibet regions (Figure 4C). Approximately 38 ASVs were identified as Actinobacteriota in each sample; however, 87 Actinobacteriota ASVs were found in the Tibet region samples. Actinobacteriota (Supplementary Table S15), one of the characteristic taxa of soil microorganisms, was previously considered to originate entirely from soil in studies of root microbiomes (Lundberg et al., 2012). Figure 4D shows that the five most abundant Actinobacteria ASVs (ASV_1457, ASV_32683, ASV_38118, ASV_39715, and ASV_37919) in scions from the Tibet region were all identified as belonging to the genus *Geodermatophilus*. A sequence comparison with the soil bacterial strains in the mulberry orchard showed that ASV_1457, ASV_32683, and ASV_38118 had identities below 98%, whereas ASV_39715 and ASV_37919 showed identities above 98%. We also paid particular attention to the two ASVs (ASV_39715 and ASV_37919) that maintained high abundance in both Tibet and non-Tibet (Supplementary Table S16). These ASVs were also found in the soil. The homology of the 5 top ASVs in the scions from non-Tibet region, respectively, was more than 98%. The results suggested that ASV_1457, ASV_32683, and ASV_38118 were possibly from Tibet, China.

**Figure 5:**
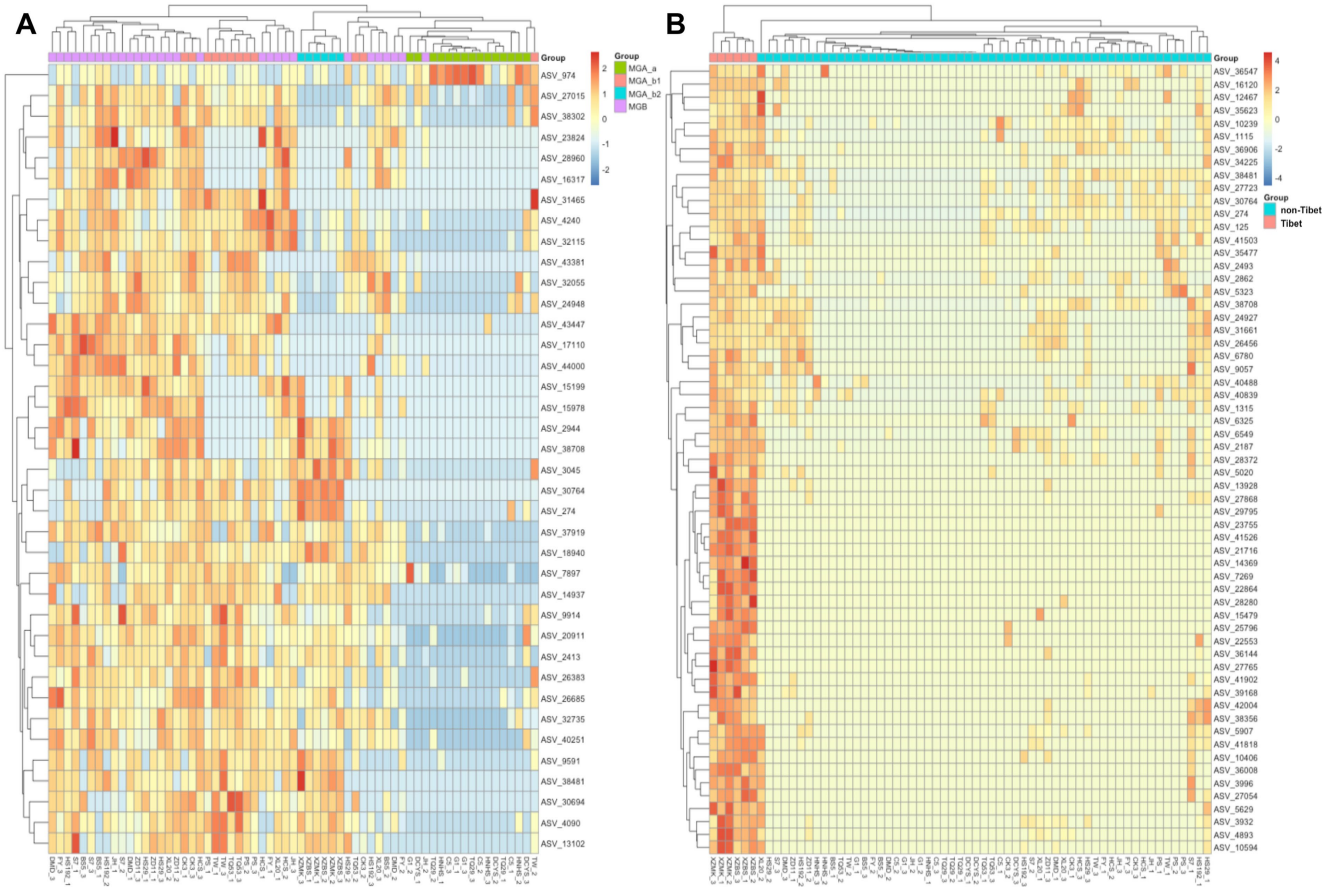
Heatmap of differentially abundant microbial features identified by ALDEx2 analysis. The heatmap displays the centered log-ratio (clr) transformed abundance of microbial taxa that were significantly different between groups. In **(A)**, statistical significance was determined using the Kruskal-Wallis test with FDR-adjusted *p*-values < 0.05. In **(B)**, significance was determined based on Welch’s *t*-test and an effect size threshold of (|effect size| > 1). Rows represented differentially abundant ASVs, and columns represented samples grouped by treatment.

The ALDEx2 analysis identified a total of 37 significantly different ASVs among the four subgroups, primarily affiliated with *Sphingomonas*, MMR, *Pseudomonas*, *Aureimonas*, and *Quadrisphaera* (Figure 5A; Supplementary Table S17). ASV_974 exhibited significantly higher abundance in the MGA_a group compared to the others. In addition, most ASVs showed higher abundance in the MGB group but lower abundance in MGA_a, suggesting that these ASVs were key features contributing to the observed intergroup differences in abundance. Samples from MGA_a and MGB largely formed two distinct clusters, with a small number of samples from MGA_b1 scattered between them. All samples from MGA_b2 formed a separate cluster. This pattern indicated a pronounced compositional divergence between MGA_a and MGB, while MGA_b1 may represent an intermediate state or exhibit high intra-group heterogeneity. In contrast, MGA_b2 appeared to possess a relatively distinct community composition. Furthermore, LEfSe analysis based on regional grouping identified 59 significantly different ASVs, primarily including members of *Sphingomonas* and *Methylobacterium-Methylorubrum* within Alphaproteobacteria, as well as ASVs affiliated with Actinobacteriota (Figure 5B; Supplementary Table S18). These findings were consistent with the taxonomic-level results of the LEfSe analysis and indicated that these ASVs contributed to the separation between the Tibet and non-Tibet groups.

### Network structure

**Figure 6:**
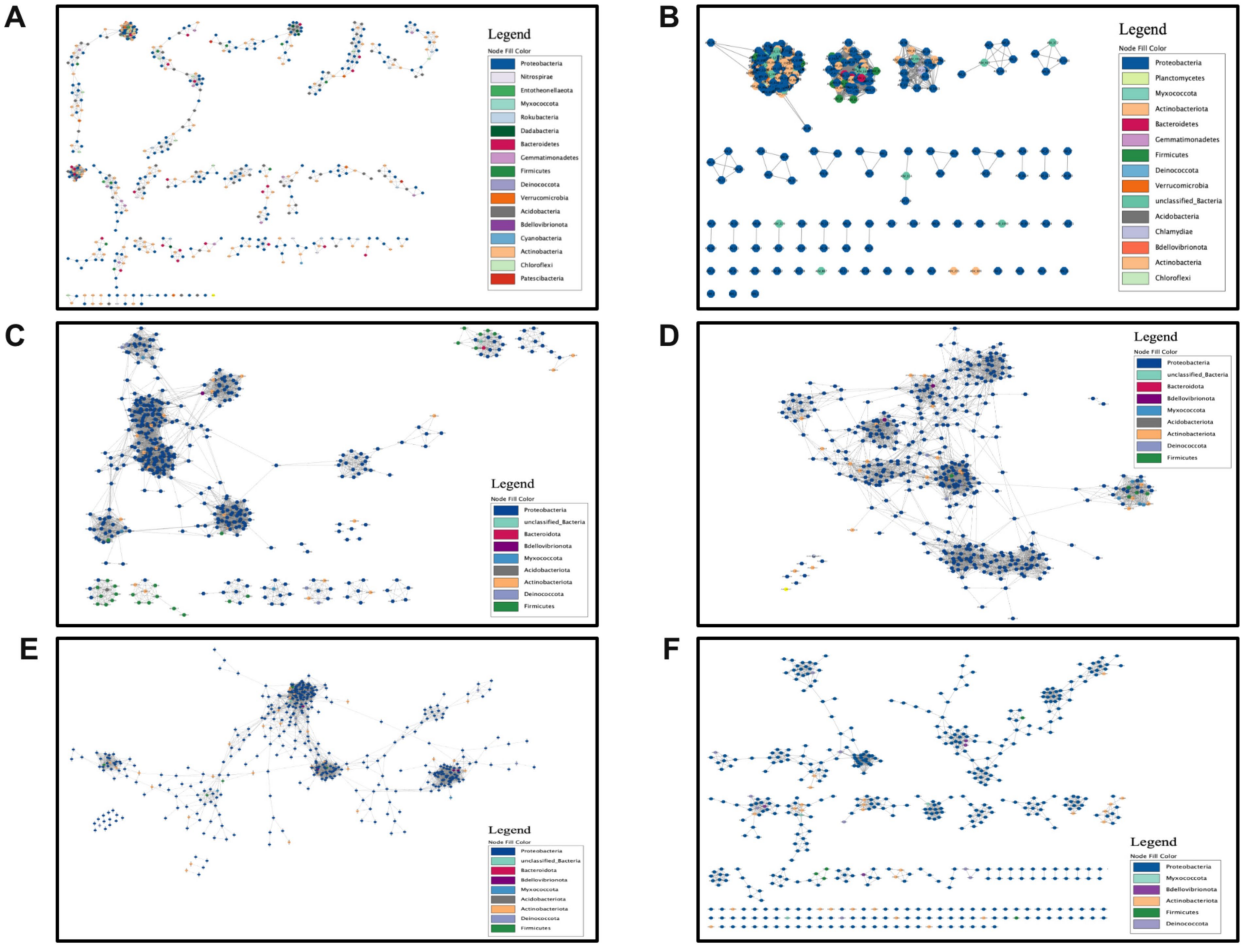
Microbial co-occurrence network. Pearson correlation coefficient threshold = 0.8, *p*-value = 0.01. **(A)** Soil. **(B)** Rootstock root. **(C)** MGA_a. **(D)** MGA_b1. **(E)** MGA_b2. **(F)** MGB. Each node represents an ASV. The network layout was generated using a force-directed algorithm implemented in Cytoscape, where the relative distances between nodes are algorithmically determined and do not directly indicate ecological or statistical relationships.

To investigate the interaction within the endophytic bacterial communities, a microbial co-occurrence network analysis was conducted, and the topological properties were calculated. The results were shown in Figure 6 and Table 2. In most networks, ASVs from Proteobacteria were the primary node components, except for the soil. In the four branch groups, some small network modules with distinct phylum-level preferences were still observed. For example, Firmicutes were prominent in MGA_a; Myxococcota and Firmicutes in MGA_b1; Actinobacteriota and Bdellovibrionota in MGA_b2; and Actinobacteriota in MGB. At the phylum level, the soil and rootstock root communities encompassed a greater diversity of phyla involved in the network composition compared to the scion branches. Furthermore, the co-occurrence networks of wild hosts (Figures 6C–E) included more phyla than those of the domesticated hosts (Figure 6F). From soil to rootstock roots to branches, the dominance of Proteobacteria in the communities gradually became more pronounced, while phyla such as Actinobacteriota, Acidobacteria, and Bacteroidetes, which were prevalent in the soil, were gradually replaced. Figure 6B displayed that the rootstock root microbial community co-occurrence network was the most complex, with an edge number of 60,788 and an average degree of 243.15, both the highest among all groups. The three groups of wild mulberry trees (edges of MGA_a = 8,037, edges of MGA_b1 = 4,775, edges of MGA_b2 = 6,767) had more edges and average degree than the domesticated mulberry group (edges of MGB = 1,394). In all samples, positive edges exceeded negative edges, with the soil samples having a higher number of negative edges (802 negative edges). We examined the connectivity of the networks for each sample. None of the networks were fully connected, indicating the presence of locally clustered substructures within the microbial communities. Therefore, we calculated the average shortest path length and network diameter of the largest connected subgraph. The root samples exhibited the lowest values for both metrics, indicating a smaller network span with fewer intermediate nodes required for connections between individuals within the communities, indicating a more compact network structure and tighter associations among taxa. Additionally, the modularity of the endophytic bacterial networks in the branches and soil was significantly higher than that in the roots, with MGB exhibiting the highest modularity. These small community modules were more prevalent in the branches and soil, but the internal connections within the modules in the roots were the tightest. In the branches, intermediate ASVs linked the modules, whereas in the soil and roots, no ASVs connected the modules, rendering them more independent.

**Table 2:** Basic network structural parameters.

Network parameters	MGA_a	MGA_b1	MGA_b2	MGB	Root	Soil
Number of nodes	443	410	448	500	500	500
Number of edges	8,037	4,775	6,767	1,394	60,788	2,663
Average degree	36.2844	23.2927	30.209	5.576	243.152	10.652
Average clustering coefficient	0.8517	0.7134	0.6889	0.5007	0.8877	0.7465
Average shortest path length	3.8363	4.6177	7.0665	5.8043	1.0116	5.1852
Diameter	12	12	21	15	4	15
Modularity	0.7071	0.8012	0.6181	0.9181	0.0467	0.7453
Number of positive edges	8,036	4,768	6,744	1,394	60,768	1861
Number of negative edges	1	7	23	0	20	802
Sparsity (1-density)	0.9179	0.9430	0.9324	0.9888	0.5127	0.9786

Based on the endophytic bacterial communities of the four branch groups, we calculated Eigenvector Centrality and Closeness Centrality to identify the ASVs closest to the key nodes in each network. Additionally, to determine the ASVs serving as connector nodes between modules in the branches, Betweenness Centrality was calculated, and the top five ASVs were highlighted. The taxa of potential key nodes and ASVs acting as bridge nodes were summarized (Supplementary Table S19). *Sphingomonas*, MMR, and *Aureimonas* were the most highly connected taxa in the network. Other taxa, such as *Deinococcus*, *Roseomonas*, ANPR, and *Frigoribacterium*, were also involved. The ASVs between modules in the branches were mainly *Sphingomonas*, MMR, and *Aureimonas*. Non-dominant taxa, including *Pseudomonas* and *Brevundimonas*, were also identified as bridge nodes between modules.

### Predicted functional profiles of endophytic bacteria in branches

**Figure 7:**
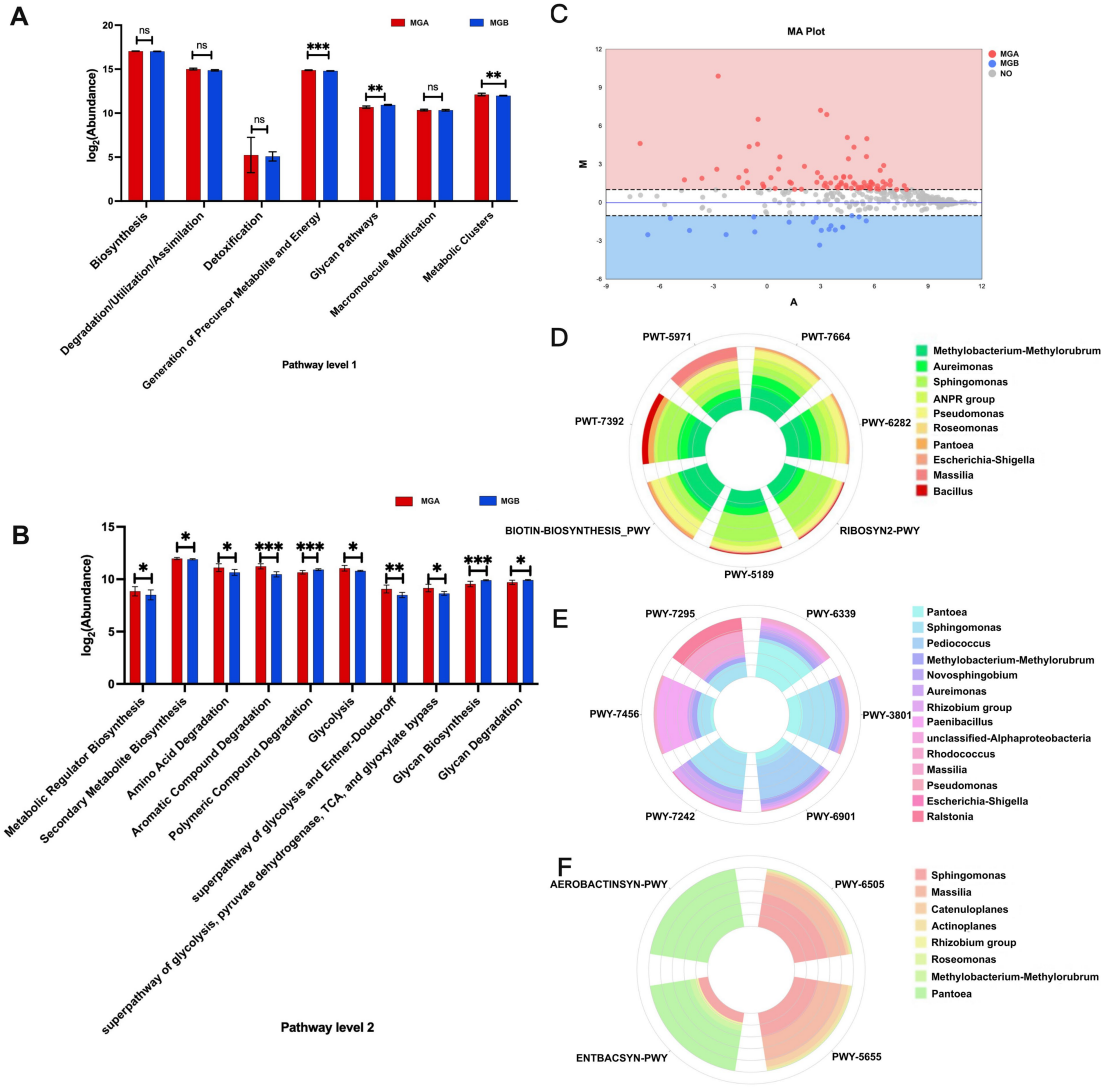
Functional prediction analysis of microbial communities. **(A)** Pathway level 1. **(B)** Pathway level 2. Significance levels: ns = not significant, * *p* < 0.05, ** *p* < 0.01, *** *p* < 0.001. **(C)** MA-plot (M-versus-A plot) was used to visualize metabolic pathways with a fold change greater than 2 in functional abundance between groups MGA and MGB. $M=\mathrm{lo}g_{2}(\mathrm{MGA})-\mathrm{lo}g_{2}(\mathrm{MGB}),$ $A=\frac{1}{2}(\mathrm{lo}g_{2}(\mathrm{MGA})+\mathrm{lo}g_{2}(\mathrm{MGB}))$ . **(D)** Represents the species composition of biomass synthesis, **(E)** represents the species composition of plant cell wall degradation, and **(F)** represents the species composition of iron acquisition, auxin biosynthesis.

Using PICRUSt2 and referencing the MetaCyc database, metabolic pathways and functions were predicted. Based on the statistical analysis of predicted abundances, Level 1 and Level 2 metabolic pathways were compared between wild and domesticated mulberry trees At pathway Level 1 (Figure 7A), seven types of metabolic functions were classified, among which three primary functions showed significant differences: Generation of Precursor Metabolite and Energy (0.0001 < *p* ≤ 0.001), Glycan Pathways (0.001 < *p* ≤ 0.01), and Metabolic Clusters (0.001 < *p* ≤ 0.01). At pathway Level 2 (Figure 7B), ten significantly different metabolic pathways were further analyzed. MGA exhibited significantly higher abundance in seven secondary functions: Metabolic Regulator Biosynthesis (0.01 < *p* ≤ 0.05), Secondary Metabolite Biosynthesis (0.01 < *p* ≤ 0.05), Amino Acid Degradation (0.01 < *p* ≤ 0.05), Aromatic Compound Degradation (0.0001 < *p* ≤ 0.001), Glycolysis (0.01 < *p* ≤ 0.05), superpathway of glycolysis and Entner-Doudoroff (0.001 < *p* ≤ 0.01), and the superpathway of glycolysis, pyruvate dehydrogenase, TCA, and glyoxylate bypass (0.01 < *p* ≤ 0.05). In contrast, Polymeric Compound Degradation (0.0001 < *p* ≤ 0.001), Glycan Biosynthesis (0.0001 < *p* ≤ 0.001), and Glycan Degradation (0.01 < *p* ≤ 0.05) were more abundant in MGB.

To further explore specific differences in metabolic pathways, the metabolic pathway abundance of endophytic bacterial communities between wild and cultivated hosts was characterized based on EC-normalized, non-hierarchical data (Figure 7C). Among the 406 predicted pathways, a total of 109 exhibited a twofold or greater difference in abundance, with 90 pathways showing higher abundance in MGA and 19 in MGB. Further analysis focused on pathways with the most pronounced differences between the two groups. The abundance of dTDP-N-acetylviosamine biosynthesis (PWY-7316), mevalonate pathway I (PWY-922), and the superpathway of geranylgeranyldiphosphate biosynthesis I (PWY-5910) in MGA exceeded that in MGB by several hundred times. In contrast, the superpathway of glycol metabolism and degradation (GLYCOL-GLYOXDEG-PWY) was the most enriched pathway in MGB, with approximately ten times the abundance relative to MGA. Additionally, the superpathway of mycolyl-arabinogalactan-peptidoglycan complex biosynthesis (PWY-6404) was annotated exclusively in MGA.

Seven pathways related to biomass synthesis were selected in Figure 7D, including oleate biosynthesis (PWY-7664), palmitoleate biosynthesis (PWY-6282), palmitate biosynthesis II (PWY-5971), taxadiene biosynthesis (PWY-7392), biotin biosynthesis (BIOTIN-BIOSYNTHESIS-PWY), flavin biosynthesis (RIBOSYN2-PWY) and tetrapyrrole biosynthesis (PWY-5189). MMR, *Aureimonas*, and *Sphingomonas* were the primary contributors. *Pseudomonas* exhibited higher abundance in biotin synthesis. MMR and *Sphingomonas* exhibited higher abundance in taxadiene biosynthesis. *Sphingomonas* had also high abundance in tetrapyrrole biosynthesis, and flavin biosynthesis I. Six pathways related to plant cell wall degradation were selected in Figure 7E including L-arabinose degradation IV (PWY-7295), syringate degradation (PWY-6339), sucrose degradation II (PWY-3801), glucose and xylose degradation (PWY-6901), mannan degradation (PWY-7456), D-fructuronate degradation (PWY-7242). *Pantoea* had higher abundance in syringate degradation, *Sphingomonas* had higher abundance in sucrose degradation II and D-fructuronate degradation. *Paenibacillus* was the primary contributor to mannan degradation. *Pediococcus* dominated the superpathway of glucose and xylose degradation. *Massilia* and *Ralstonia* were notable in L-arabinose degradation IV. Four pathways related to auxin biosynthesis and iron acquisition were selected in Figure 7F. *Pantoea* made significant contributions in the pathways of aerobactin biosynthesis (AEROBACTINSYN-PWY) and enterobactin biosynthesis (ENTBACSYN-PWY).

### Host genetics is associated with microbiome composition

**Figure 8:**
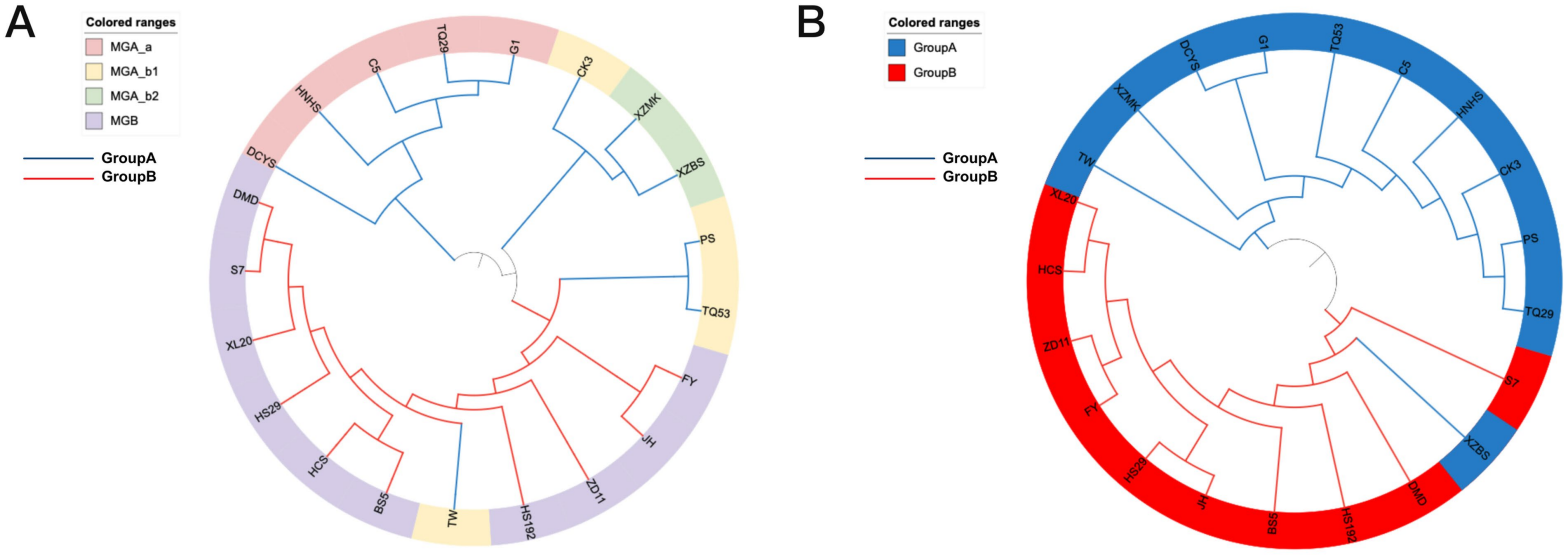
Community structure of endophytic bacteria and phylogenetic relationships of mulberry hosts. **(A)** Community tree of endophytic bacterial communities constructed based on unweighted UniFrac distances. **(B)** Phylogenetic tree of mulberry genetic resources constructed based on host single nucleotide polymorphism (SNP) variations. The colored ranges represent different sample groupings, and branch colors indicate the grouping of host plants.

To further determine the association between host genotype and endophytic bacterial community characteristics, 21 mulberry germplasm resources were sequenced by a simplified genome sequencing approach. A phylogenetic tree was constructed based on single nucleotide polymorphism (SNP) mutation sites (Figure 8A; Supplementary Figure S5). The mulberry germplasm resources were grouped into two major branches: the 10 wild resources (except XZBS) clustered together, while the cultivated resources formed another distinct cluster. At the same time, hierarchical clustering analysis of endophytic bacterial community composition was also performed, and sample similarity was visualized in a dendrogram based on unweighted UniFrac distance (Figure 8B). The endophytic bacterial communities of MGA samples clustered together, except for TW, while those of MGB also formed a distinct cluster. The hierarchical clustering of endophytic bacterial communities was largely consistent with the host phylogenetic tree.

## Discussion

### Dominant taxa and ecological insights

The present study provides comprehensive insights into the diversity, functional potential, and host-genotype association of endophytic bacterial communities in grafted mulberry (*Morus* spp.). Our findings reveal that Proteobacteria dominate the endophytic microbiome in mulberry branches (89.07%). This prominent dominance of Proteobacteria is consistent with previous studies on endophytic bacterial diversity in mulberry branches (Ou et al., 2019). In addition, Proteobacteria are more amenable to *in vitro* cultivation and isolation (Xu et al., 2019), which facilitates the investigation of their metabolic versatility and symbiotic potential. As the habitat transitioned from soil to rootstocks and then to branches, the relative abundance of Proteobacteria increased, whereas that of soil-dominant Actinobacteriota and Acidobacteriota declined, indicating the root system’s role as an effective biological filter (Edwards et al., 2015). At the genus level, *Sphingomonas* and MMR were dominant in most samples, consistent with the findings of Yu et al. in the study of *Stevia rebaudiana* Bertoni leaves (Yu et al., 2015). Notably, *Sphingomonas* exhibited the most pronounced stepwise enrichment across the three habitats, ultimately achieving the highest relative abundance in branches. This enrichment pattern was similar to the observations reported by Chen et al. regarding endophytic bacterial communities in mulberry roots and branches (Chen et al., 2022). *Sphingomonas* strains are known to produce auxin, nitric oxide, and siderophores, exhibit ACC deaminase activity, and promote the growth of certain *Brassicaceae* plants (Mazoyon et al., 2023).

### Diversity and drivers of endophytic bacterial communities in mulberry

**Figure 9:**
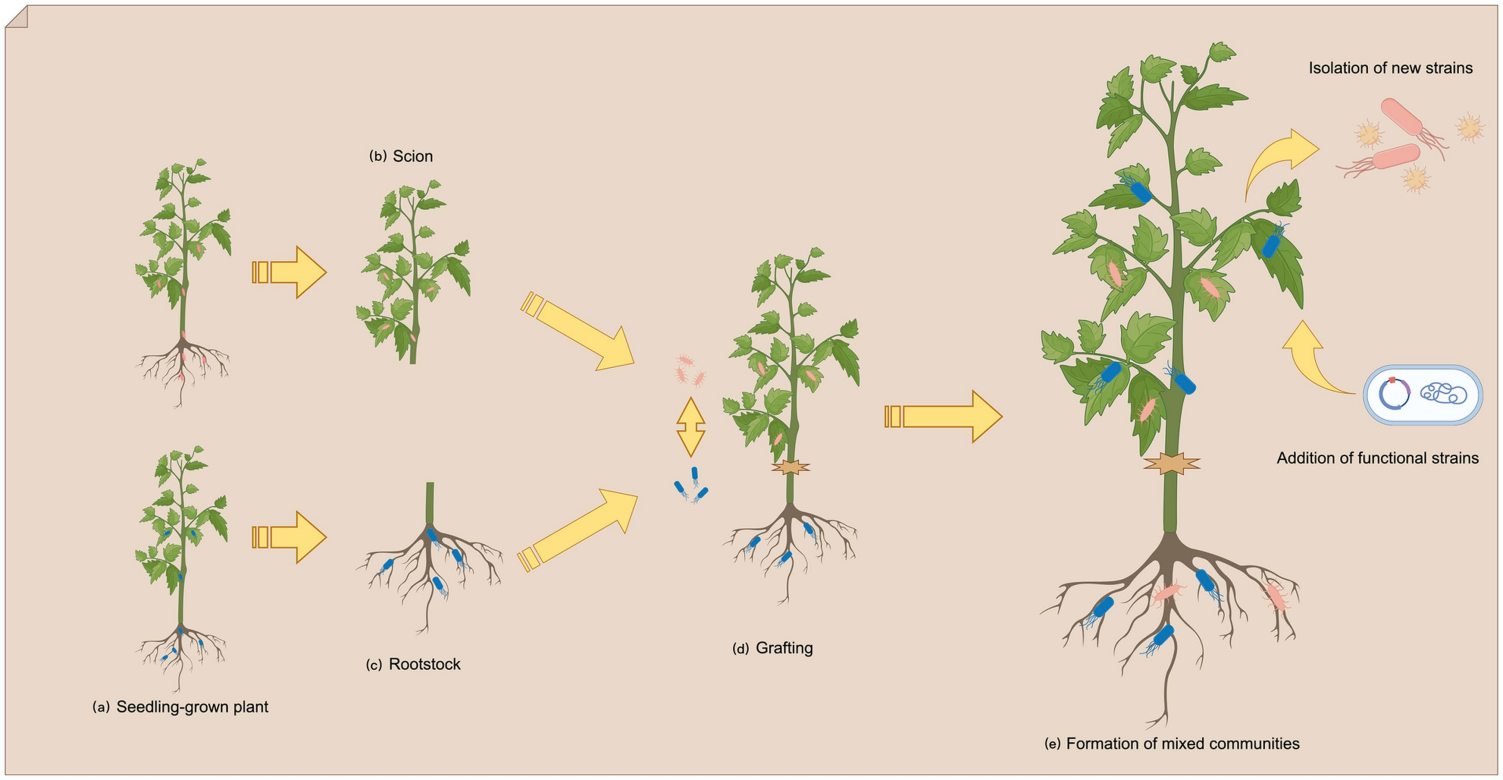
Diagrammatic of endophytic bacteria origin by grafting propagation **(a)** Plants grown from seeds develop under different environmental conditions. **(b)** Scion-associated microbial community. **(c)** Rootstock-associated microbial community. **(d)** Grafting combines different scions and rootstocks. **(e)** A new mixed microbial community is formed.

The composition of plant endophytic bacterial communities is significantly influenced by soil physicochemical properties and agricultural management practices, including fertilization (Lin et al., 2024; Zhong et al., 2024), tillage methods (Tyler, 2019; Sui et al., 2024), and pesticide application (Ma et al., 2023). Soil acts as a primary reservoir for these microorganisms (Philippot et al., 2013), facilitating bacterial colonization through the root interface. Meanwhile, the host genotype further modulates community structure (Ali et al., 2021; Vergine et al., 2024). Unlike most experimental systems where the “root–branch” continuum shares a uniform genotype, mulberry is typically cultivated through grafting, whereby genetically distinct scions and rootstocks are combined and grow in differing soil environments. Grafting has been employed to enhance the environmental adaptability of scions by using stress-resistant rootstocks (Jin et al., 2023), a practice that induces microbial community differentiation between roots and branches during early plant development (Figure 9). In perennial mulberry trees, one-year-old branches can be regarded as annual structures, as they are routinely pruned during summer to rejuvenate the tree, optimize canopy structure, and facilitate harvesting (Kaushal et al., 2019). From the summer pruning in May to the following pruning season, newly developed mulberry shoots typically grow from dormant winter buds into fully lignified branches within approximately 3 months. This developmental process, which encompasses the period from bud break to lignification, occurs over a short time span and closely overlaps with seasonal changes. Previous studies have demonstrated that the dynamics of endophytic bacterial communities are tightly linked to host growth stages (Wei et al., 2024). Rapid tissue expansion during early development provides new ecological niches for microbial colonization, while transpiration-driven xylem flow promotes the internal movement of microbial communities (Compant et al., 2005; Frank et al., 2017). A study on *Nitraria tangutorum* found that microbial competition within the endophytic community weakens progressively as plant lignification advances, ultimately leading to a more stable community structure (Kang et al., 2023). Based on these findings, we infer that the endophytic microbial communities in newly developed mulberry branches reach a relatively stable dynamic equilibrium once structural maturity is achieved. Accordingly, sampling was performed prior to the subsequent summer pruning, ensuring that each branch had undergone a complete growth cycle, and that microbial competition associated with rapid growth had subsided. This strategy enabled us to characterize the diversity of mulberry-associated endophytic bacterial communities during a relatively stable developmental phase. In this study, the 21 mulberry germplasm resources were classified into four groups based on the diversity of their endophytic bacterial communities. Among them, MGA_a, MGA_b1, and MGB had been domesticated for over 18 years, whereas the samples in MGA_b2 had undergone only 5 years of domestication. In the two Tibetan mulberry samples of MGA_b2, Actinobacteria were identified as representative biomarkers. Previous studies in *Arabidopsis thaliana* have shown that Actinobacteria are often derived from the soil in which the plant grows (Lundberg et al., 2012; Bhatti et al., 2017). Several ASVs belonging to the *Geodermatophilus* were exclusively detected in MGA_b2 and could not be matched with high similarity to ASVs present in the soil samples. *Geodermatophilus* is recognized as an extremophilic microorganism capable of surviving harsh environmental conditions and exhibiting strong adaptability and diverse metabolic capabilities (Demirjian et al., 2001; Essoussi et al., 2010). The separation of MGA_b2 from other groups in terms of community diversity and biomarker taxa is likely attributed to both the unique geographic origin of the Qinghai–Tibet Plateau and the shorter duration of graft-induced domestication. These findings suggest that scions, as vegetative propagules, may retain part of their original endophytic microbiota established during early development, even at the time of mulberry germplasm collection. This legacy effect may be one of the underlying reasons for the observed diversity preservation in scion-associated microbial communities under identical grafting conditions. Based on the potential micro-ecological community assembly patterns influenced by this legacy effect, we propose a model for the application of endophytic bacteria to improve crop traits in grafted plants such as mulberry, which are commonly propagated through grafting in agricultural practice. In this model, functional bacterial strains or synthetic microbial communities could be artificially introduced during the early stages of scion cultivation under controlled conditions. By concentrating the inoculation on the scions, these microbes may persist over the long term in the grafted shoots and exert their beneficial functions within a more stable internal environment. This strategy could also avoid the disruption of soil microbial communities often associated with the direct application of microbial agents to soil, thereby reducing the risk of excessive accumulation of exogenous microbes in the rhizosphere. After all, many plant-associated endophytic bacteria may also possess potential pathogenicity to humans (Nithya and Babu, 2017). By artificially manipulating, coordinating, or preconditioning the endophytic microbial community during crop development, it is possible to maintain a healthy and balanced microbial state. Such regulation can enhance the beneficial roles of endophytic plant bacteria (EPB) within the community and guide the overall functional trajectory of the microbiome toward a mutually beneficial relationship with the host plant.

### Variations between wild and cultivated mulberry resources

Our results demonstrate that wild mulberry genotypes harbor more diverse and complex endophytic bacterial communities than their cultivated counterparts. First, the genetic distances among samples within Group A were relatively large, likely resulting in divergent host-driven selection pressures (Marques et al., 2015). Second, the original ecological environments of wild germplasm were more heterogeneous than those of agricultural lands (Paula et al., 2014), while anthropogenic activities associated with crop production impose stronger selective pressures on soil microbial communities. In contrast, the MGB group exhibited lower intra-group variation and greater community similarity, possibly due to the constraint of agricultural soil environments shaping microbial assemblages across cultivated resources. Additionally, the lower evenness observed in MGB communities may be partially attributed to the dominance of *Sphingomonas*. Variation in the copy number of the 16S rRNA gene may also have contributed to the observed differences in microbial community composition (Gao and Wu, 2023). Venn diagram analyses reflected similar trends at the taxonomic level: due to the broad origins and greater genetic divergence of MGA members, the number of shared ASVs was notably lower. Among them, the MGA_a subgroup exhibited the lowest species richness and the greatest degree of community differentiation, whereas MGA_b1 displayed the highest microbial diversity and a community composition more similar to that of MGB. This similarity may be associated with a higher degree of host domestication within MGA_b1. These findings support the hypothesis that domestication reduces microbial diversity, potentially as a consequence of breeding programs prioritizing yield-related traits at the expense of traits associated with microbiome-mediated resilience (Porter and Sachs, 2020). Moreover, the strong correlation between host SNP-based phylogeny and microbial community composition further highlights the genotype-dependent assembly of endophytic bacteria—an observation consistent with recent findings in the tomato symbiotic microbiome (Li et al., 2024). However, we encountered challenges in quantitatively linking host SNP data with bacterial community variation, which may be due to limitations in sample size and the mismatch in the scale of variation between host genotypes and microbiomes.

### Co-occurrence network and functional prediction

In the co-occurrence network from soil to roots to branches, the dominant components of the network gradually changed. Proteobacteria were the dominant component in the network of mulberry one-year-old branches, serving as both key nodes and hubs—a finding consistent with the previous research by Ou et al. (2019). Based on the average degree and average clustering coefficient, the complexity of the root network was the highest, with the internal connections between nodes significantly greater than those in the shoot microbiome, and even higher than in the soil. This contrasts with the results from Chao Xiong et al., who found that the complexity of soil communities was much higher than that of root microbiomes in their analysis of field crop soil and root microbial networks (Xiong et al., 2021). We speculated that this result might be related to the diversified organic secretions of the roots (Bais et al., 2006; Jones, 2009). Under the influence of associated metabolic pathways, the correlations between microorganisms were amplified. Joseph Edwards constructed a network for the CH_4_ cycling of rice root microbiomes and identified 15 modules containing methanogenic OTUs (Edwards et al., 2015).

Functional prediction was one of our primary concerns. During symbiosis with the host, endophytic bacteria exhibit diverse functional characteristics due to their metabolic properties. These functions include promoting nutrient absorption (Santoyo et al., 2016), enhancing resistance to adverse environments (Wang et al., 2021), antagonizing pathogenic microorganisms (Ahmed et al., 2023), and participating in the co-secretion of plant secondary metabolites (Zhang et al., 2020; Zheng et al., 2023). These functions influenced research priorities in plant endophytic bacterial studies and provided a practical basis for utilizing these beneficial microorganisms. The greater diversity of endophytic bacteria in wild mulberry trees, together with functional prediction results, suggests that wild hosts may have greater potential for screening functionally beneficial strains compared to domesticated hosts. In our study, we focused on four key metabolic functions: synthesis of specific functional metabolites, plant cell wall degradation, auxin biosynthesis, and iron acquisition. Among the various mechanisms by which endophytic bacteria directly promote plant growth, auxin biosynthesis represents a key cooperative strategy with the host. For instance, in tomato roots, seven strains with indole-3-acetic acid (IAA) production capacity and plant growth-promoting effects have been isolated. Some of these strains possess complete biosynthetic pathways such as the indole-3-acetamide (IAM) and tryptamine (TAM) pathways, while others can synthesize IAA only by utilizing intermediate compounds derived from the host (Feng et al., 2024). In studies on chrysanthemum tissue culture, endophytic bacteria exhibited a clear growth trend on media supplemented with high concentrations of cytokinins (5–20 μM) (Panicker et al., 2007). These findings suggest that phytohormones play a crucial role in mediating interactions between endophytic bacteria and their plant hosts and may underlie the long-term cooperative relationships observed in such associations. In the present study, we identified several bacterial genera with high predicted functional abundance, including *Sphingomonas*, *Pantoea*, and *Pseudomonas*. These genera will serve as key targets for future isolation and functional investigation. *Pantoea* contributed to all of the aforementioned functions, and studies have demonstrated its significant potential in biological nitrogen fixation as well as strong environmental adaptability. Its diverse plant growth-promoting capabilities may reflect a more intimate cooperative relationship with host plants, making it a strong candidate for the development of biofertilizers and the implementation of sustainable agricultural practices (Loiret et al., 2004). By optimizing culture media conditions—such as pH, nitrogen sources, and carbon sources—it may be possible to selectively enhance their recovery. Moreover, these microbes may be developed as model systems for studying the mechanisms by which endophytic bacteria establish cooperative relationships with plants. However, we still lack a clear understanding of the relationship between microbial abundance and the actual, observable functional expression of these bacteria.

## Conclusion

This study employed 16S rRNA high-throughput sequencing to systematically elucidate the characteristic features and functional potential of endophytic bacterial communities in one-year-old mulberry (*Morus* spp.) branches. By examining community variations across wild and cultivated mulberry genotypes, the study identified host genetic distance and grafting as key factors influencing community similarity. These findings underscore the role of domestication in reducing microbial diversity and highlight the influence of grafting in restructuring microbial community assembly effects that are particularly pronounced in perennial crops. The diverse endophytic microbiota associated with wild mulberry exhibited higher functional potential, while the convergent communities in cultivated varieties reflected the selective pressures and co-adaptive processes shaped by human-mediated domestication. This research significantly advances our understanding of the relationship between host genetic background and endophytic bacterial community composition, providing a foundation for the targeted exploration and application of mulberry-associated microbiota. Importantly, the study specifically considered grafting as an agricultural technique and its impact on endophytic bacterial variation, offering practical insights relevant to real-world cultivation systems. Future studies should focus on field-based validation and high-throughput culturing approaches to bridge the gap between predicted and expressed microbial functions, thereby promoting the practical use of mulberry endophytes in sustainable agricultural practices.

## Data Availability

The original contributions presented in the study are publicly available. This data can be found here: https://www.ncbi.nlm.nih.gov/, accession number: PRJNA1255390.

## Footnotes

^1^ https://docs.qiime2.org/2019.4/tutorials/

^2^ http://greengenes.secondgenome.com/

^3^ http://www.microbesonline.org/fasttree/

^4^ https://metacyc.org/

^5^ https://www.kegg.jp/

^6^ https://www.ncbi.nlm.nih.gov/COG/

^7^ https://www.genescloud.cn

^8^ https://blast.ncbi.nlm.nih.gov

^9^ https://itol.embl.de/

^10^ https://cnsknowall.com

^11^ https://www.figdraw.com
