# Supplementary material for: Diversity of endophytic bacteria in mulberry (Morus spp.) scions with different genetic resources
Source: Front Microbiol. 2025 Jun 24;16:1618773. doi: 10.3389/fmicb.2025.1618773 (PMC12237256; doi:10.3389/fmicb.2025.1618773)
Supplement: Supplementary file 2 [file Supplementary_file_2.docx]

Supplementary Material

| **Table S7 Sequence Information** | | | |
| --- | --- | --- | --- |
| Sample | Number of Sequences | Total Number of Bases | Average Read Length |
| Soil | 263145 | 110131088 | 418.5186418 |
| Root | 268611 | 100763011 | 375.1261527 |
| Branch | 4023912 | 1516090966 | 376.770408 |

| **Table S8 Phylum Species Composition** | | | | | | |
| --- | --- | --- | --- | --- | --- | --- |
| Phylum | | Soil (Relative Abundance) | | Root (Relative Abundance) | Branch (Relative Abundance) | |
| *Proteobacteria* | 0.423106 | | 0.835163 | | | 0.8927 |
| *Actinobacteria* | 0.243409 | | 0.117288 | | | 0.030591 |
| *Firmicutes* | 0.017023 | | 0.012503 | | | 0.050098 |
| *Bacteroidetes* | 0.04656 | | 0.006257 | | | 0.000555 |
| *Acidobacteria* | 0.122494 | | 0.003743 | | | 0.000509 |
| *Chloroflexi* | 0.040563 | | 0.002277 | | | 0 |
| *Chlamydiae* | 0 | | 0.004332 | | | 0 |
| *Myxococcota* | 0 | | 0 | | | 0.00374 |
| *Deinococcota* | 0 | | 0 | | | 0.018481 |
| Others | 0.106844 | | 0.018436 | | | 0.003325 |

| **Table S9 Phylum Species Composition of Branch** | | | | | |  |  |  |  |  |  |
| --- | --- | --- | --- | --- | --- | --- | --- | --- | --- | --- | --- |
| Phylum | MGA (Relative Abundance) | | | | | | MGB (Relative Abundance) | | |  |  |
| *Proteobacteria* | 0.85150905 | | | | | | 0.93389143 | | |  |  |
| *Firmicutes* | 0.08912503 | | | | | | 0.01107055 | | |  |  |
| *Actinobacteriota* | 0.04014186 | | | | | | 0.02104087 | | |  |  |
| *Deinococcota* | 0.00686949 | | | | | | 0.03009322 | | |  |  |
| *Myxococcota* | 0.00644447 | | | | | | 0.00103644 | | |  |  |
| *Bdellovibrionota* | 0.00211297 | | | | | | 0.00135847 | | |  |  |
| *Bacteroidota* | 0.00107955 | | | | | | 0.0000313 | | |  |  |
| *Acidobacteriota* | 0.00100078 | | | | | | 0.0000169 | | |  |  |
| *Gemmatimonadota* | 0.0000568 | | | | | | 0.0000288 | | |  |  |
| *Planctomycetota* | 0.0000641 | | | | | | 0 | | |  |  |
| Others | 0.00159582 | | | | | | 0.00143202 | | |  |  |
|  |  | | | | | |  | | |  |  |
| **Table S10 Genus Species Composition** | | | | | | | | | | | |
| Genus | | | Soil (Relative Abundance) | | | Root (Relative Abundance) | | | | Branch (Relative Abundance) | |
| *Sphingomonas* | | | 0.02294977 | | 0.019572 | | | | 0.333511 | | |
| MMR | | | | 0 | 0 | | | | 0.18621 | | |
| *Aureimonas* | | | | 0 | 0 | | | | 0.087404 | | |
| *Pseudomonas* | | | | 0 | 0.027197 | | | | 0.05963 | | |
| ANPR | | | | 0 | 0 | | | | 0.056758 | | |
| Subgroup_6 | | | | 0.06373436 | 0 | | | | 0 | | |
| *Mycobacterium* | | | | 0.03375999 | 0 | | | | 0 | | |
| *Steroidobacter* | | | | 0.03049668 | 0 | | | | 0 | | |
| *Bradyrhizobium* | | | | 0.02796738 | 0 | | | | 0 | | |
| *Streptomyces* | | | | 0.02793836 | 0 | | | | 0 | | |
| *Ralstonia* | | | | 0 | 0.351298 | | | | 0 | | |
| *Delftia* | | | | 0 | 0.106664 | | | | 0 | | |
| *Stenotrophomonas* | | | | 0 | 0.063411 | | | | 0 | | |
| Others | | | | 0.79315346 | 0.431859 | | | | 0.276486 | | |

| **Table S11 Genus Species Composition of Branch** | | | | |  | |
| --- | --- | --- | --- | --- | --- | --- |
| Genus | | MGA | | MGB | |  |
| *Sphingomonas* | 0.22703266 | | 0.43999025 | | |  |
| *Methylobacterium-Methylorubrum* | 0.1901819 | | 0.18223854 | | |  |
| *Aureimonas* | 0.09083514 | | 0.08397338 | | |  |
| *Pseudomonas* | 0.08521653 | | 0.03404402 | | |  |
| *Allorhizobium-Neorhizobium-Pararhizobium-Rhizobium* | 0.05816319 | | 0.05535186 | | |  |
| *Pediococcus* | 0.05030683 | | 0.00006708 | | |  |
| *Deinococcus* | 0.00686949 | | 0.03008697 | | |  |
| *Pantoea* | 0.0039195 | | 0.02712195 | | |  |
| *Roseomonas* | 0.02075085 | | 0.00943585 | | |  |
| *Massilia* | 0.01539668 | | 0.008207 | | |  |
| Others | 0.25132723 | | 0.12948312 | | |  |

| **Table S12 The 21 Samples Shared ASVs.** | | |  |
| --- | --- | --- | --- |
| ID | Genus | Average abundance | |
| >ASV_43798 | Methylobacterium-Methylorubrum | 3139.571429 | |
| >ASV_124 | Aureimonas | 2017.238095 | |
| >ASV_42255 | Sphingomonas | 3449.698413 | |
| >ASV_16605 | Allorhizobium-Neorhizobium-Pararhizobium-Rhizobium | 1812.253968 | |
| >ASV_28370 | Methylobacterium-Methylorubrum | 1216.68254 | |
| >ASV_22161 | Pseudomonas | 148.2380952 | |
| >ASV_15300 | Rubrobacter | 27.32258065 | |
| >ASV_9072 | Pseudomonas | 5.714285714 | |

| **Table S16 The top ten most abundant Actinobacteria phylum taxa.** | |
| --- | --- |
| non-Tibet | Tibet |
| ASV_33148 | ASV_1457 |
| ASV_10512 | ASV_1315 |
| ASV_37919 | ASV_39715 |
| ASV_24477 | ASV_25368 |
| ASV_1428 | ASV_32683 |
| ASV_39715 | ASV_37919 |
| ASV_41588 | ASV_13091 |
| ASV_35181 | ASV_38118 |
| ASV_10882 | ASV_11193 |
| ASV_4152 | ASV_37677 |

| **Table S19 Centrality indicators** | | | | | | | |
| --- | --- | --- | --- | --- | --- | --- | --- |
| Sample | Betweenness Centrality | | Eigenvector Centrality | | Closeness Centrality | | |
| MGA_a | ASV_18958 | Sphingomonas | ASV_23282 | MMR | ASV_23282 | MMR |  |
|  | ASV_14370 | Sphingomonas | ASV_14937 | Aureimonas | ASV_14937 | Aureimonas |  |
|  | ASV_27119 | Pseudomonas | ASV_22514 | Sphingomonas | ASV_26038 | Sphingomonas |  |
|  | ASV_24948 | unclassified_Rhizobiales | ASV_38481 | MMR | ASV_28476 | Aureimonas |  |
|  | ASV_41631 | unclassified_Alphaproteobacteria | ASV_30006 | MMR | ASV_22514 | Sphingomonas |  |
| MGA_b1 | ASV_40839 | MMR | ASV_14865 | Sphingomonas | ASV_6058 | MMR |  |
|  | ASV_25125 | MMR | ASV_8757 | unclassified_Beijerinckiaceae | ASV_26904 | unclassified_Rhizobiales |  |
|  | ASV_26499 | Sphingomonas | ASV_34132 | Deinococcus | ASV_20324 | Aureimonas |  |
|  | ASV_5451 | Brevundimonas | ASV_42648 | Roseomonas | ASV_13117 | Roseomonas |  |
|  | ASV_18030 | Sphingomonas | ASV_24477 | Frigoribacterium | ASV_20097 | Aureimonas |  |
| MGA_b2 | ASV_4567 | Sphingomonas | ASV_23199 | MMR | ASV_7938 | Sphingomonas |  |
|  | ASV_4090 | Sphingomonas | ASV_27065 | Aureimonas | ASV_40561 | unclassified_Comamonadaceae |  |
|  | ASV_20324 | Aureimonas | ASV_26864 | ANPR | ASV_18381 | Aureimonas |  |
|  | ASV_6435 | Sphingomonas | ASV_17724 | unclassified_Rhizobiaceae | ASV_4138 | Sphingomonas |  |
|  | ASV_31902 | unclassified_Beijerinckiaceae | ASV_8534 | Sphingomonas | ASV_12349 | Roseomonas |  |
| MGB | ASV_10708 | Deinococcus | ASV_2656 | MMR | ASV_10708 | Deinococcus |  |
|  | ASV_24530 | Sphingomonas | ASV_4219 | MMR | ASV_24530 | Sphingomonas |  |
|  | ASV_32109 | Aureimonas | ASV_22351 | Sphingomonas | ASV_5363 | Aureimonas |  |
|  | ASV_20324 | Aureimonas | ASV_36547 | Sphingomonas | ASV_32109 | Aureimonas |  |
|  | ASV_8163 | unclassified_Alphaproteobacteria | ASV_17098 | MMR | ASV_719 | ANPR |  |

**Figure S1 Rarefaction curves**

In the figure, the x-axis represents the rarefaction depth, while the y-axis displays the median Chao1 index from ten iterations along with box plots. In the box plots, the components are defined as follows: the upper and lower edges of the box indicate the interquartile range (IQR); the central line within the box represents the median; and the whiskers denote the maximum and minimum values within 1.5 times the IQR. (A)Soil sample. (B)Root sample. (C)Branch sample.


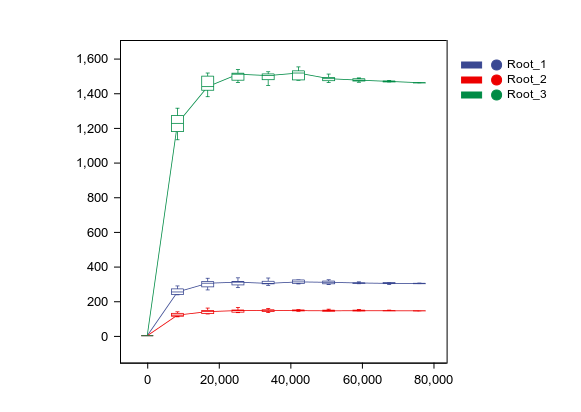

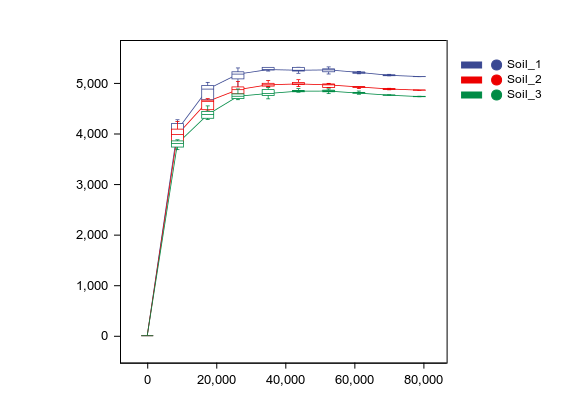

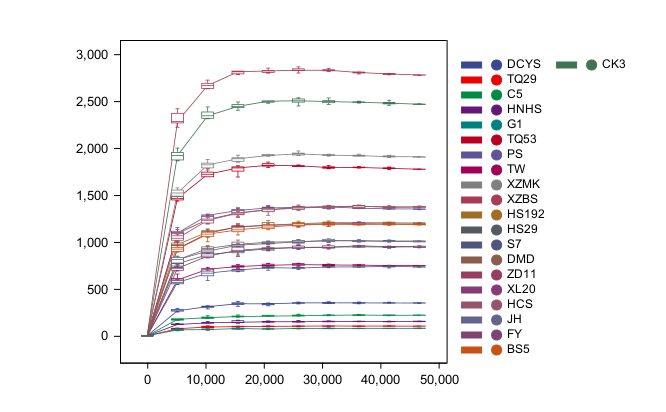


A

B

C

**
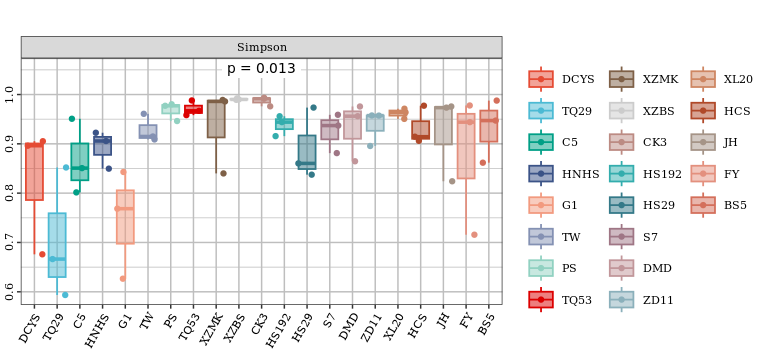

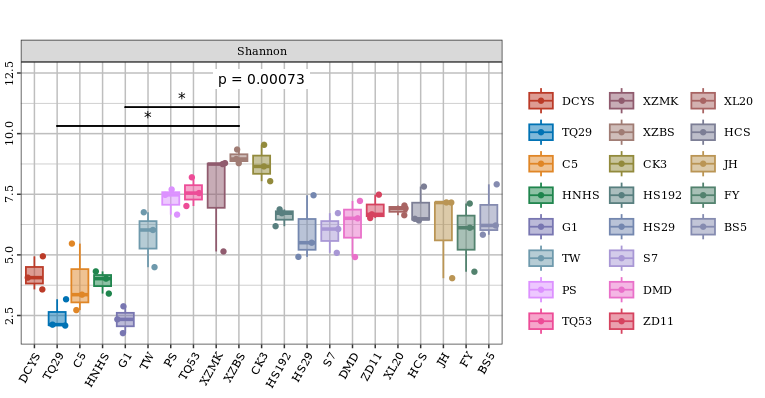

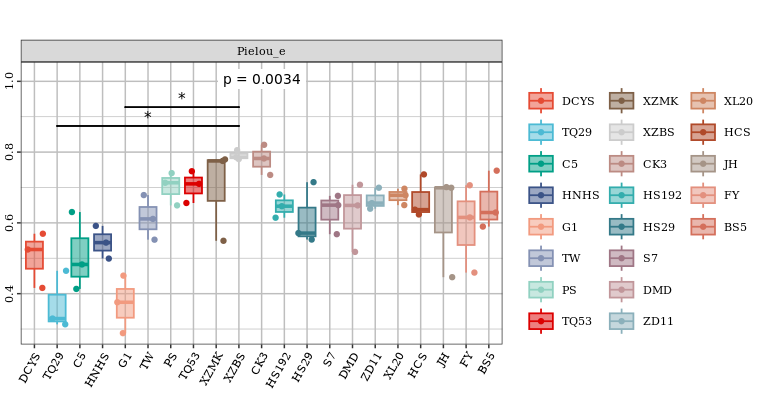

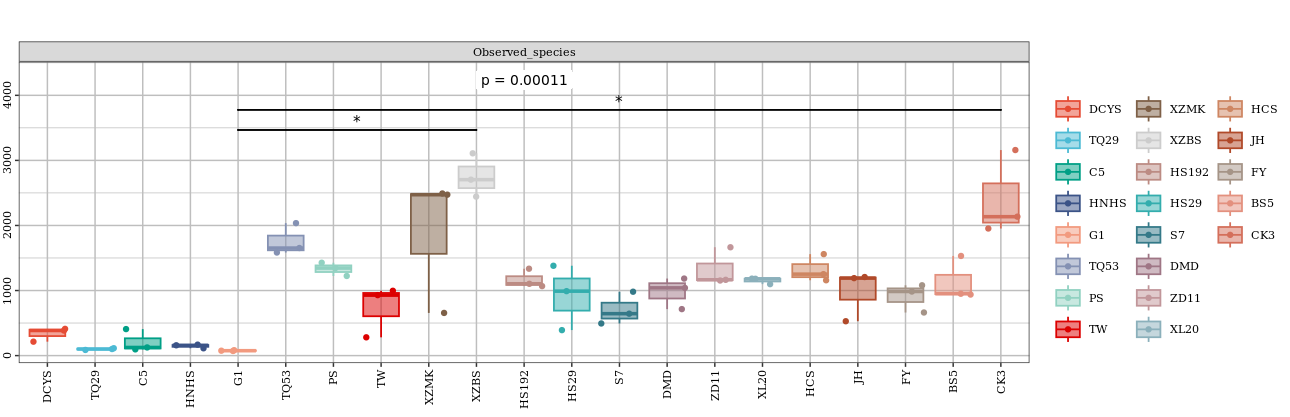

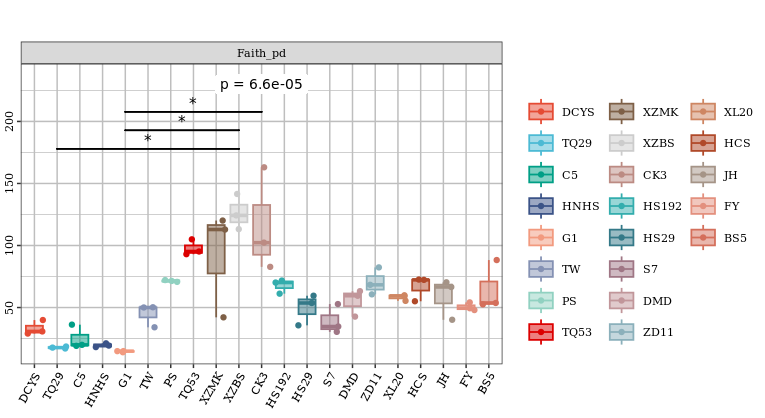

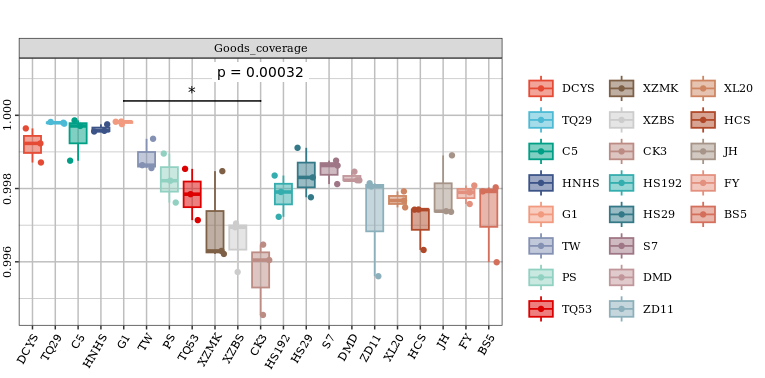

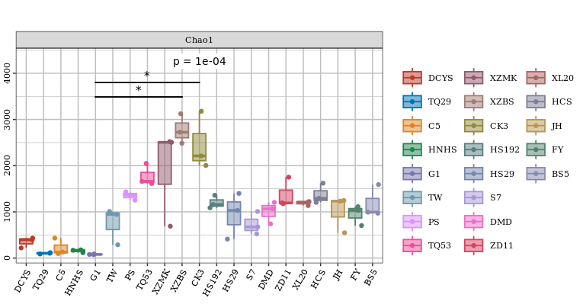
Figure S2 alpha diversity index of Branches**

F

G

E

D

C

B

A

In the figure, the x-axis represents group labels, and the y-axis indicates the corresponding alpha diversity index values. In the box plots, the components are defined as follows: the upper and lower edges of the box represent the interquartile range (IQR); the line inside the box indicates the median; the whiskers show the maximum and minimum values within 1.5 times the IQR; and the dots outside the whiskers represent outliers. The numbers below the diversity index labels denote the p-values from the Kruskal-Wallis test. (A)Chao1. (B) Faith’s pd. (C) Goods coverage. (D) Observed Species. (E) Pielou’s evenness. (F) Shannon. (G) Simpson.

**
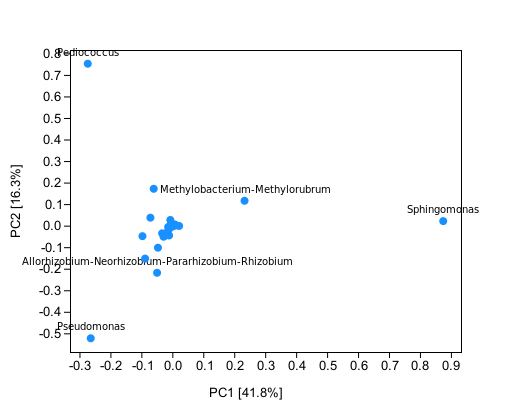
Fihure S3 Loading Plot**

In the figure, each point represents a species (default: genus). The x- and y-coordinates of each point can be interpreted as the extent to which the species contributes to sample variation along the corresponding dimensions. The percentages in parentheses on each axis indicate the proportion of total variance in species abundance composition explained by that dimension across all samples. The top five contributors were labeled with their corresponding genus names.


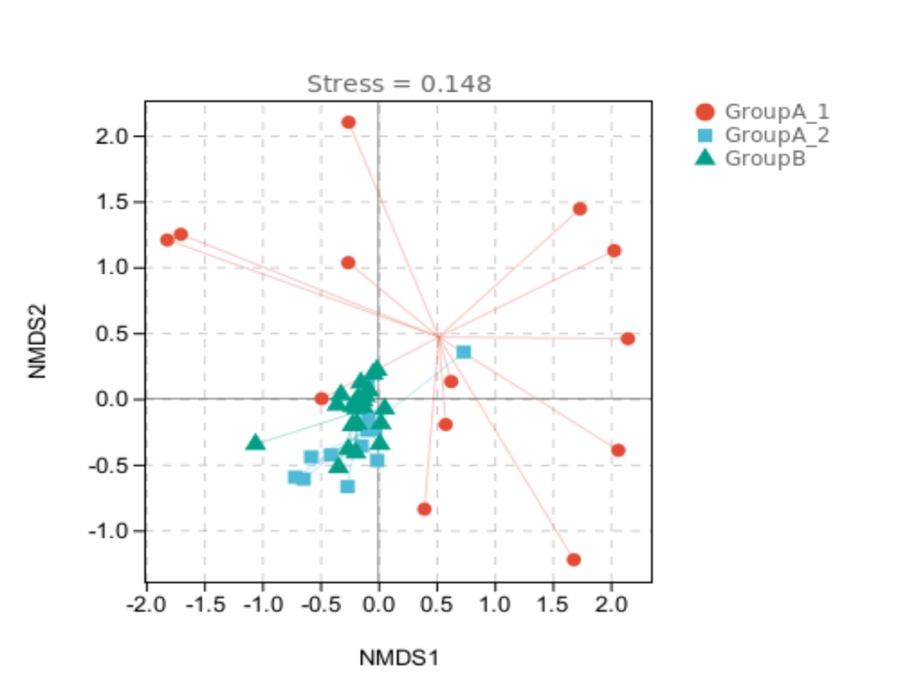


**Figure S4 NMDS analysis (weighted UniFrac)**

Each point in the figure represents a sample, with different colors indicating different groups. NMDS is based on rank orders; therefore, the closer (or farther) the distance between two points, the smaller (or larger) the difference in microbial community composition between the corresponding samples. The weighted UniFrac distance algorithm was used in the analysis.

**
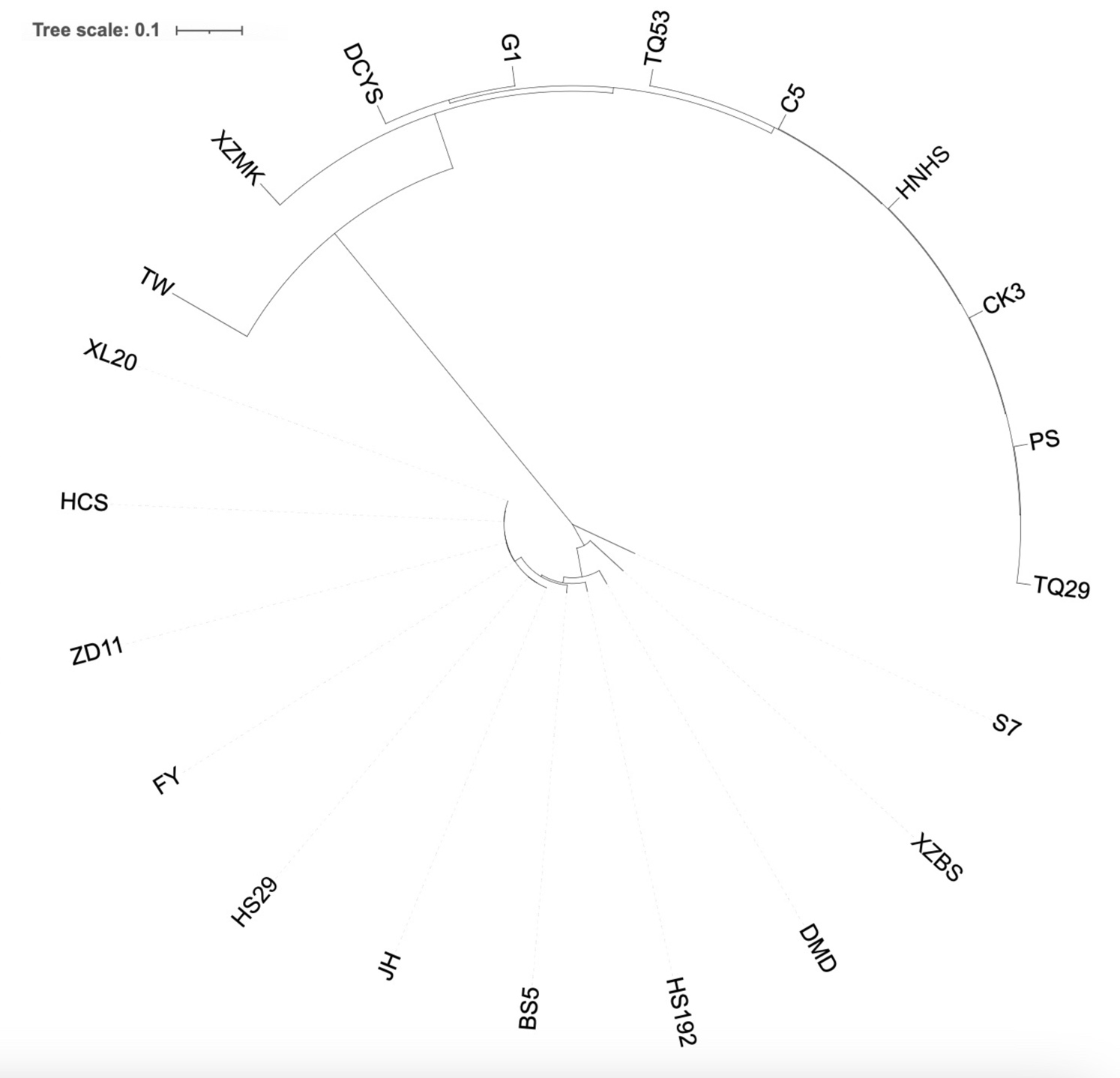
**

**Figure S5 Phylogenetic tree of 21 mulberry samples**

**
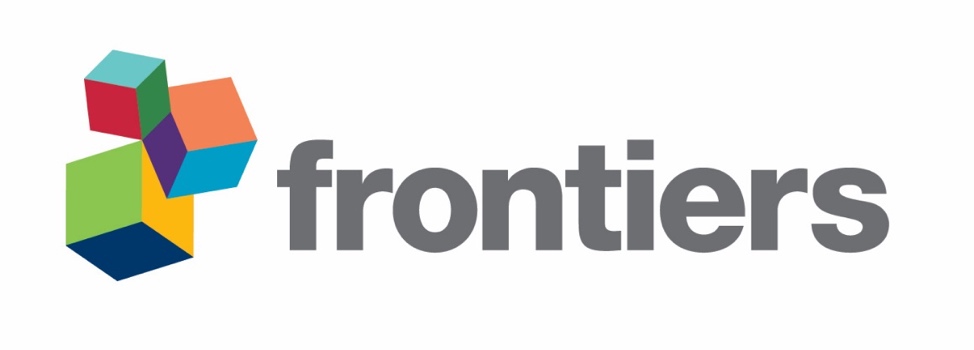
**
